# Genetic Association Between Polymyositis/Dermatomyositis and Epilepsy: Insights From Mendelian Randomization and Bioinformatic Analyses

**DOI:** 10.1002/brb3.71148

**Published:** 2025-12-29

**Authors:** Ying Liu, Yuhang Yu, Chulong Fang, Qingzhong Wu, Lingli Ou, Jianhao Xiao, Kui Duan, Ting Jiang, Lan Ye, Xiao Hu, Zhanhui Feng

**Affiliations:** ^1^ Department of Neurology Affiliated Hospital of Guizhou Medical University Guiyang Guizhou China; ^2^ Department of Clinical Medicine, School of Clinical Medicine Guizhou Medical University Guiyang Guizhou China; ^3^ Department of Anesthesiology The Second Affiliated Hospital of Guizhou University of Chinese Medicine Guiyang Guizhou China; ^4^ Department of Pharmacology, School of Basic Medical Science Guizhou Medical University Guiyang Guizhou China; ^5^ Department of Neurology Guizhou Provincial People's Hospital Guiyang Guizhou China

**Keywords:** dermatomyositis, epilepsy, immune cell infiltration, Mendelian randomization, polymyositis, single‐cell transcriptomics

## Abstract

**Background:**

Idiopathic inflammatory myositis (IIM), comprising polymyositis (PM) and dermatomyositis (DM), is a collective term for immune‐mediated diseases characterized by skeletal muscle inflammation. Emerging evidence points to an increased incidence of epilepsy in patients with PM/DM. However, the causality and underlying mechanisms behind this association are unclear. Our study aimed to explore the potential causal link between PM/DM and epilepsy, with a focus on immune‐mediated mechanisms, using Mendelian randomization (MR) and transcriptome analyses.

**Methods:**

Initially, summary data from genome‐wide association studies (GWAS) related to polymyositis (PM; finn‐b‐M13_POLYMYO), dermatomyositis (DM; finn‐b‐DERMATOPOLY_FG), and epilepsy (ebi‐a‐GCST90018840) were obtained from the Integrative Epidemiology Unit Open GWAS database. These data were utilized for Mendelian randomization (MR) analysis and generalized summary data based Mendelian randomization (GSMR). To ensure the robustness of the findings, sensitivity analyses were conducted to corroborate the results of the MR analyses. Subsequently, the study leveraged publicly accessible databases and bioinformatics tools to conduct comprehensive analyses of gene expression data. This included differential expression analysis, immune infiltration analysis, and gene enrichment analysis. Differentially expressed SNP‐related genes (DE‐SRGs) were further analyzed using single‐cell transcriptomics. Finally, the expression of four key genes (IER3, TNF, GPANK1, and ATF6B) in the hippocampus of epilepsy mouse model was quantified using PCR.

**Results:**

The MR analysis disclosed a causal association between PM and epilepsy, whereas the reverse MR analysis did not identify a significant causal effect of epilepsy on PM. However, there was no association between DM and epilepsy of MR analysis. The Transcriptome analysis not only identified DE‐SRGs but also revealed distinct immune cell infiltration patterns in epilepsy patients. Specifically, we observed SRGs are mainly expressed in endothelial cells, microglia, and T cells, indicative of a proinflammatory state. Furthermore, the gene set variation analysis (GSVA) highlighted the differential activation of pathways in these cell types, including inflammatory response and allograft rejection, which were significantly upregulated. PCR results show the expression of IER3, TNF, GPANK1, and ATF6B in hippocampus of epilepsy model largely consistent with bioinformatics predictions.

**Conclusion:**

The study reveals a causal association between PM and epilepsy, with no significant impact of epilepsy on PM. There is no causal association between DM and epilepsy. The absence of a DM‐epilepsy link may reflect fundamental differences in immunopathology: while PM is driven by T cell–mediated muscle invasion, DM involves predominant humoral immunity and complement deposition, suggesting distinct neuroinflammatory implications. Our findings establish immune‐mediated neuroinflammation as the central mechanistic link between PM and epileptogenesis. These findings implicate shared immunopathogenic mechanisms and suggest therapeutic targets for epilepsy associated with polymyositis.

## Graphical abstract

This Mendelian randomization study provides robust evidence for a causal link between polymyositis (PM) and epilepsy, highlighting the role of immune‐mediated mechanisms in epilepsy development, while no such association was found for dermatomyositis (DM). Through bioinformatic and statistical analyses, key genes and pathways were identified, with IER3, TNF, GPANK1, and ATF6B emerging as central functional factors.

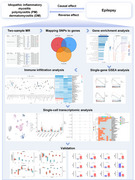

**Abbreviations**
ATF6B: activating transcription factor 6 beta
BP: biological processes
CC: cellular components
CI: confidence interval
CNS: central nervous system
DEGs: differentially expressed genes
DE‐SRGs: differentially expressed SNP‐related genes
DM: dermatomyositis
GO: Gene Ontology
GPANK1: G‐patch domain and ankyrin repeats 1
GSMR: generalized summary data–based Mendelian randomization
GSVA: gene set variation analysis
GWAS: genome‐wide association study
IER3: immediate early response 3
IIM: idiopathic inflammatory myositis
IVs: Instrumental variables
IVW: inverse variance‐weighted
KEGG: Kyoto Encyclopedia of Genes and Genomes
LD: linkage disequilibrium
MF: molecular functions
MR: Mendelian randomization
NF‐κb: nuclear factor kappa‐light‐chain‐enhancer of activated B cells
OR: odds ratio
PM: polymyositis
SNPs: single nucleotide polymorphisms
SRGs: SNP‐related genes
ssGSEA: single sample Gene Set Enrichment Analysis
TNFα: tumor necrosis factor alpha
UMI: unique molecular identifier

## 1 Introduction

Idiopathic inflammatory myositis (IIM) represents a diverse group of immune‐mediated disorders, primarily defined by chronic inflammation of the skeletal muscles (Lundberg et al. 2021). Among adults, polymyositis (PM) and dermatomyositis (DM) are the predominant clinical subtypes of IIM (Dalakas 2015; Lilleker et al. 2018). These conditions are typically marked by the subacute development of symmetrical weakness in proximal muscles. Additionally, they often involve other organ systems, such as the lungs and skin. They are also strongly associated with the presence of autoantibodies and tend to respond well to immunosuppressive therapies. The etiology of PM and DM is complex and multifactorial, involving genetic predispositions, immune dysregulation, and environmental triggers (Miller et al. 2018).

A cross‐sectional study has indicated that individuals with polymyositis (PM) exhibit a higher incidence of epilepsy compared to control populations (Nissan et al. 2021). Nevertheless, the causal relationship between PM and epilepsy remains uncertain and requires further investigation. Epilepsy is a common and highly debilitating chronic disorder of the central nervous system (CNS), characterized by sudden abnormal neuronal discharges in the brain. It affects over 70 million people worldwide (Devinsky et al. 2018; Thijs et al. 2019; Keezer et al. 2016; Erlangsen et al. 2020). The etiology and pathogenesis of epilepsy are highly complex, encompassing genetic, structural, metabolic, immune, infectious, and idiopathic factors (Scheffer et al. 2017). A growing body of evidence suggests an autoimmune etiology for some forms of epilepsy, supported by a documented clinical overlap between epilepsy and autoimmune disorders (Amanat et al. 2019). As Steriade et al. (2021) highlight, systemic immune dysfunction can lower seizure thresholds through innate immune activation, blood–brain barrier (BBB) disruption, and adaptive immune responses. Notably, immune cell infiltration is a hallmark of the epileptic brain, with studies demonstrating the presence of B lymphocytes, T lymphocytes, and natural killer (NK) cells in the epileptic tissues of humans and animal models. The migration of these lymphocytes into the central nervous system (CNS) is believed to disrupt the BBB and accelerate epileptogenesis (Ravizza et al. 2008; Fabene et al. 2008; Hildebrandt et al. 2008; Xu et al. 2013). This immune dysregulation is a shared feature of idiopathic inflammatory myopathies (IIM) and epilepsy, indicating potential mechanistic crosstalk. In polymyositis and dermatomyositis (PM/DM), immune abnormalities coincide with elevated levels of proinflammatory cytokines (e.g., TNF‐α, IL‐1β, IL‐6) and chemokines, which can compromise BBB integrity (Shinjo et al. 2013; Steriade et al. 2021). Once impaired, the BBB permits circulating immune cells (e.g., CD8+ T cells, B cells) and inflammatory mediators to infiltrate the brain parenchyma. Within the CNS, these cytokines are known to alter neuronal excitability, synaptic function, and glial activation, thereby directly promoting seizure generation and recurrence (Steriade et al. 2021).

Mendelian randomization (MR) addresses limitations of observational studies (e.g., confounding, reverse causation) by using genetically determined variants as instrumental variables, enabling robust causal inference (Dobrijevic et al. 2023; Gu et al. 2023). Complementary multi‐omics approaches such as single‐cell RNA sequencing (scRNA‐seq) can dissect cellular heterogeneity and functional states (Hernández Martínez et al. 2022; Wu et al. 2023), while bioinformatics analysis of differentially expressed genes (DEGs) in epileptic brain tissues has identified immune responses, inflammation, and cytokine signaling as key pathways (He et al. 2023). Integrating these tools allows simultaneous investigation of causality and underlying mechanisms.

Therefore, the primary aim of this study is to explore the potential causal relationship between PM/DM and epilepsy through Mendelian Randomization analysis and other multi‐omics approaches. Additionally, this study seeks to elucidate the biological mechanisms and microenvironmental changes by which PM/DM may influence epilepsy progression.

## 2 Methods

### 2.1 Study Design

**FIGURE 1:**
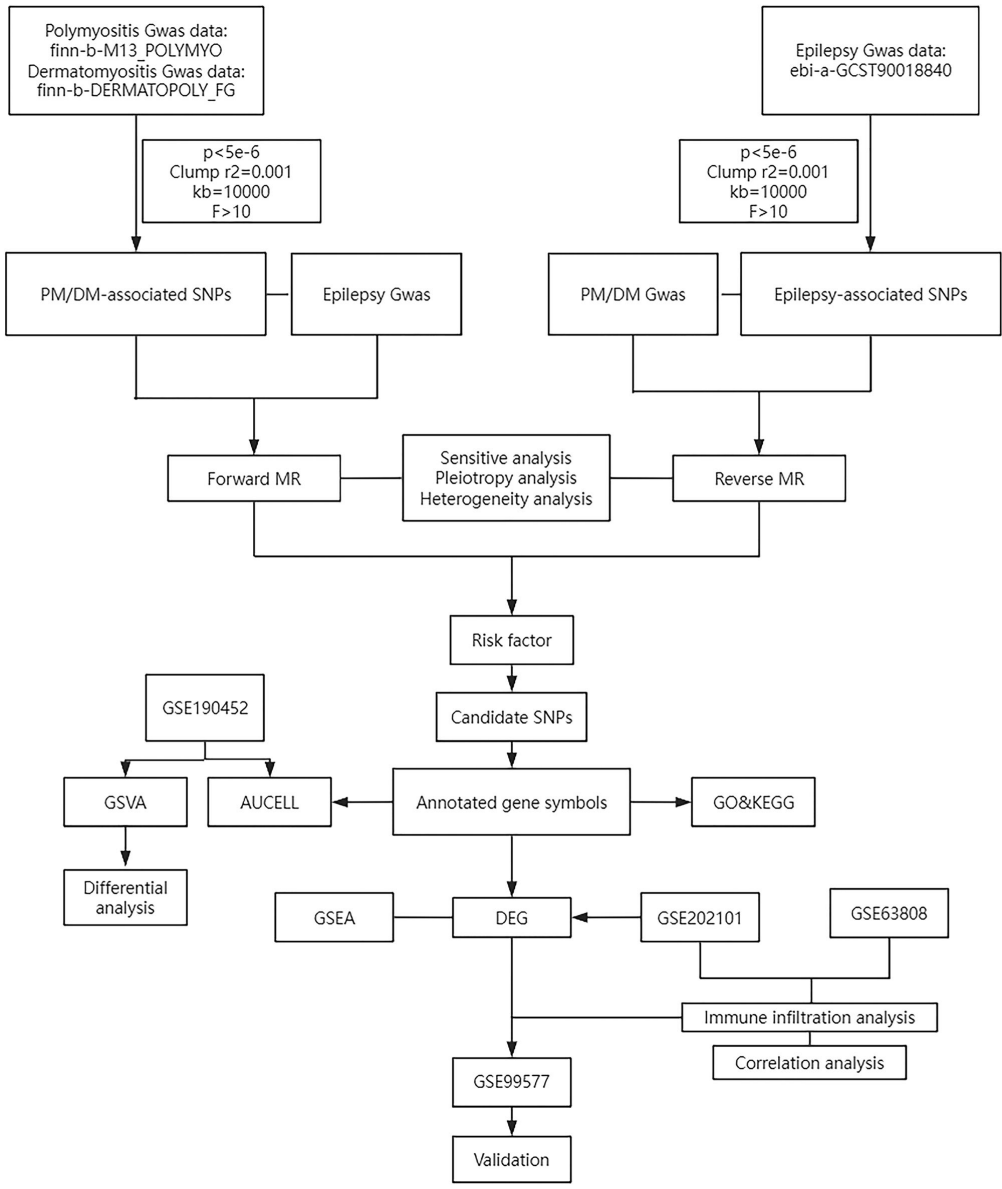
The workflow of the analyses.

Firstly, two‐sample MR was employed to assess the causal relationship between PM/DM and epilepsy, the robustness of the MR findings was corroborated through generalized summary data–based Mendelian randomization (GSMR). The study methodology complied with the requirements in the STROBE‐MR checklist. In MR, genetic variants serve as proxies for modifiable exposures. For valid causal inference, these instrumental variables (IVs) must satisfy three core assumptions: (1) the genetic variants are strongly associated with the exposure of interest; (2) the genetic variants are independent of any confounding factors that may exist between the exposure and the outcome; and (3) the genetic variants influence the outcome exclusively through the exposure, rather than via alternative pathways (Davies et al. 2018). Secondly, we utilized a diverse range of publicly accessible databases to conduct an extensive analysis. This included differential expression analysis, immune infiltration analysis, and Gene Ontology (GO) analysis, aimed at elucidating the common mechanisms linking epilepsy and PM. The study workflow is illustrated in Figure 1.

### 2.2 MR Study

#### 2.2.1 GWAS Data Sources

We conducted a MR analysis using publicly available genome‐wide association study (GWAS) summary statistics from the Integrative Epidemiology Unit OpenGWAS database (https://gwas.mrcieu.ac.uk/). Data for epilepsy (ebi‐a‐GCST90018840) were sourced from a meta‐analysis of UK Biobank and FinnGen, including 458,310 individuals of European ancestry. Data for polymyositis (PM; finn‐b‐M13_POLYMYO) and dermatomyositis (DM; finn‐b‐DERMATOPOLY_FG) were sourced from the FinnGen consortium, comprising 213,264 and 173,035 European‐ancestry participants, respectively. A partial sample overlap exists between the FinnGen cohorts used for the exposures (PM/DM) and the outcome (epilepsy). All original studies had obtained informed consent and ethical approval from their respective review boards; therefore, no additional ethical clearance was required for this analysis (Kurki et al. 2023; Sakaue et al. 2021).

#### 2.2.2 Instrumental Variable (IV) Selection

To ensure the validity of our MR analysis, we applied stringent criteria for IV selection. First, we selected single‐nucleotide polymorphisms (SNPs) associated with the exposure at a genome‐wide significance threshold (*p* < 5×10^−6^) (Kwok and Schooling 2021). Second, to guarantee independence among the IVs, we performed linkage disequilibrium (LD) clumping (*r* ^2^ < 0.001 within a 10,000 kb window) using the 1000 Genomes European reference panel (Chen et al. 2022), thereby minimizing the risk of overestimating the number of independent tests. We further assessed IV strength using the proportion of variance explained (*R* ^2^) and the *F*‐statistic, calculated as follows: *R* ^2^ = 2 × beta2 × EAF × (1 – EAF)/(2 × beta2 × EAF × (1 – EAF) + SE2 × 2 × *N* × EAF(1 – EAF)), *F* = (*N* – 2) × *R* ^2^/(1 – *R* ^2^). SNPs with an *F*‐statistic below 10 were excluded to prevent weak instrument bias (Burgess and Thompson 2011). We then applied the generalized summary data–based MR (GSMR) method (Xue et al. 2024), which accounts for LD among correlated IVs and removes SNPs with potential pleiotropic effects using a heterogeneity in dependent instruments (HEIDI)‐outlier threshold of *p* < 0.01. Finally, we harmonized the SNP‐exposure and SNP‐outcome datasets, removing palindromic SNPs. To address potential reverse causation, we performed Steiger filtering (Hemani et al. 2017) on the harmonized data prior to the main analysis.

#### 2.2.3 MR Methods

This study employed a bidirectional two‐sample Mendelian randomization (MR) analysis to evaluate potential causal relationships between PM/DM and epilepsy. The analysis was performed using R software (version 4.4.1) with the “TwoSampleMR” (version 0.6.7) (Hemani et al. 2018) and “MR‐PRESSO” (version 1.0) (Verbanck et al. 2018) packages. We applied five MR methods: inverse variance‐weighted (IVW), MR–Egger, weighted median, simple mode, and weighted mode. The IVW method served as the primary analytical approach, as it provides a precise inverse variance‐weighted average of the ratio estimates from all genetic variants, assuming all are valid instrumental variables or that horizontal pleiotropy is balanced. To ensure robust causal inference and mitigate bias, we leveraged the complementary strengths of these methods. For further validation, we applied the generalized summary data–based MR (GSMR) method using the “GSMR2” (version 1.1.1) R package, which provides enhanced causal estimates that are less susceptible to bias from correlated pleiotropy (Xue et al. 2024). We treated the four bidirectional analyses as independent tests. To control the risk of false positives, a Bonferroni correction was applied, establishing a significance threshold of *p* < 0.0125 (α = 0.05/4). Additionally, we assessed potential bias from sample overlap by evaluating the type 1 error rate with an online calculator (https://sb452.shinyapps.io/overlap/) (Burgess et al. 2016).

#### 2.2.4 Sensitivity Analysis

We examined the potential variability among instrumental variables in each analysis through Cochran's *Q* test. A *p*‐value exceeding 0.05 suggests that significant heterogeneity is not present (Bowden et al. 2018). To evaluate horizontal pleiotropy, we utilized the MR–Egger intercept test (Burgess and Thompson 2017). A *p*‐value greater than 0.05 indicates that there is no evidence of horizontal pleiotropy. Additionally, we applied the Mendelian Randomization Pleiotropy Residual Sum and Outlier (MR‐PRESSO) test to identify and address horizontal pleiotropy by eliminating outliers (Verbanck et al. 2018). Although we used various methods to improve the accuracy of the instrumental variables during their evaluation, some may still introduce bias into the causal estimates. Therefore, we conducted a sensitivity analysis using the leave‐one‐out method to further confirm the reliability of our findings (Lee and Song 2019).

#### 2.2.5 Reverse MR Analysis

The identical procedure was employed for the reverse MR analysis, and IVW was utilized as the principal analytical technique.

### 2.3 Bioinformatic Analysis

#### 2.3.1 Data Source

The gene expression data for epilepsy (GSE202101 (Maurer‐Morelli et al. 2022), GSE63808 (Johnson et al. 2015), GSE99577 (Chali et al. 2019), GSE190452 (Chen et al. 2023)) was obtained from the NCBI Gene Expression Omnibus public database (the GEO database). The dataset GSE202101 includes gene expression data from 8 patients with epilepsy and 7 healthy individuals. The dataset GSE63808 includes gene expression data from 129 patients with epilepsy. The dataset GSE99577 comprises gene expression data in the CA1 region of the mouse hippocampus at specific time intervals: 6 h, 1 day, 2 days, 4 days, 6 days, and 12 days following the administration of kainic acid (KA, the KA group) or NaCl (the control group). The dataset GSE190452 includes single‐cell data from the cortex of 4 patients with epilepsy and 4 healthy individuals.

#### 2.3.2 Mapping SNPs to Genes

The investigation delved into the underlying molecular mechanisms to comprehensively assess the impact of polymyositis (PM) on epilepsy. Initially, the eQTL mapping technique was employed to annotate SNPs associated with polymyositis into corresponding gene symbols, which were identified by significant cis‐eQTLs within the eQTLGen Consortium database (https://eqtlgen.org/) and GTEx Brain region (hippocampus) eQTL data (https://www.gtexportal.org/). These annotated genes, referred to as SNP‐related genes (SRGs), were subsequently utilized for further analysis.

#### 2.3.3 Differential Expression Analysis

The dataset GSE202101 was used for differential analysis. The uploader had applied the limma method to remove batch effects before we downloaded it. We assessed the effectiveness of this correction using a box plot (Figure S5). Differential expression analysis of epilepsy and control group samples was performed using the “limma” (version 3.60.4) package (Ritchie et al. 2015). Genes with *p* < 0.05 and |logFC| > 0.585 were considered as differentially expressed genes (DEGs). Subsequently, we used the “ggvenn” (version 0.1.10) package in R to find the overlap of Differentially Expressed Genes (DEGs), SNP‐related genes (SRGs). We named these common genes as Differentially Expressed SNP‐related genes (DE‐SRGs). Subsequently, we validated the trend of DE‐SRGs using the GSE99577 dataset.

#### 2.3.4 Gene Enrichment Analysis

To elucidate the potential biological roles of the genes mapped by the IVs for polymyositis, functional enrichment analysis was conducted. The “clusterProfiler” R package (version 4.12.6) (Xu et al. 2024) was utilized to perform enrichment analysis across Gene Ontology (GO) categories, including Biological Processes (BP), Cellular Components (CC), and Molecular Functions (MF), as well as Kyoto Encyclopedia of Genes and Genomes (KEGG) pathways. A significance threshold of adjusted *p*‐value < 0.05 was established, and the top 10 most significant GO terms and pathways were graphically represented using the “ggplot2” R package. For single‐sample Gene Set Enrichment Analysis (GSEA), the samples from the GSE63808 dataset were categorized into high‐expression and low‐expression groups according to the median expression level of each differentially expressed SNP‐related gene (DE‐SRG). Differential expression analysis was carried out using the “limma” package, followed by GSEA. Terms with an adjusted *p*‐value < 0.05 were identified as significantly enriched.

#### 2.3.5 Immune Infiltration Analysis

To evaluate the infiltration of immune cells in epilepsy patients, we initially obtained 58 immune cell features from CellMarker 2.0 database (Hu et al. 2023) (Table S3). Subsequently, we employed the single‐sample Gene Set Enrichment Analysis (ssGSEA) method via the GSVA R package (version 1.38.2) (Hänzelmann et al. 2013). This method, an extension of the GSEA approach, enables the calculation of an enrichment fraction that indicates the absolute enrichment degree of a gene set in each sample within a given dataset. This fraction was used to estimate the relative abundance of each immune cell type in epilepsy patients. We utilized the GSE202101 dataset to examine the variations in immune cell infiltration among epilepsy patients and the GSE63808 dataset to explore the correlation between gene expression profiles and immune cell abundance in the context of epilepsy.

#### 2.3.6 Single‐Cell Transcriptomic Analysis

The expression levels of DE‐SRGs across various cell types in the GSE190452 dataset were evaluated using the “Seurat” R package (version 5.1.0) (Hao et al. 2024). The analysis commenced with stringent quality control measures applied to the single‐cell RNA sequencing data. Specifically, Cells with unique molecular identifier (UMI) counts below 1000 or above the 97th percentile of the dataset's UMI distribution were excluded. Additionally, cells expressing fewer than 300 or more than 7000 genes were removed. Only cells with mitochondrial gene expression accounting for less than 10% of total gene expression were retained. To identify the most variable genes, the “Find Variable Features” function with the “vst” method was employed, pinpointing the top 3000 highly variable genes. These results were visually represented through scatter plots. For comprehensive cell group evaluation and analysis, linear dimensionality reduction was conducted based on gene expression profiles using the “ElbowPlot” function. Subsequently, cell clustering was performed on the reduced expression matrix with the “FindNeighbors” and “FindClusters” functions, setting the resolution at 0.8. Cell types were annotated with reference to marker genes from prior studies. To assess the enrichment of SRGs in single‐cell RNA sequencing data, AUCell (version 1.27.0) was utilized, and the cellular localization patterns of DE‐SRGs were illustrated via bubble plots. Furthermore, gene set variation analysis (GSVA) was conducted to explore the biological functions of cell clusters in the GSE190452 dataset, leveraging “hallmark gene sets.” Differential expression analysis was then carried out using the “limma” package. Pathways with a *p*‐value less than 0.05 and an absolute log fold change greater than 1 were deemed differentially expressed.

### 2.4 Construction of Epilepsy Model

Adult male C57BL/6 mice (8–10 weeks, 20–25 g) were purchased from SPF (Beijing) Biotechnology Co., Ltd. All animals were housed under standard conditions (27°C, 55%–65% humidity, 12/12‐h light/dark cycle) with free access to food and water for one week prior to experiments. All procedures were approved by the Animal Ethics Committee of Guizhou Medical University (Approval No. 2304024) and conducted in accordance with the guidelines.

Mice were randomly assigned to control and model groups (*n* = 10 per group per model). Three distinct chemical kindling models were established as follows: (1) Pentylenetetrazol (PTZ) Kindling: Mice in the PTZ group received a daily intraperitoneal injection of PTZ 35 mg/kg (Sigma, P6500) daily for 28 days. (2) Lithium‐Pilocarpine (Li‐PC) Kindling: To establish the Li‐PC model, atropine was intraperitoneally injected at a dose of 1 mg/kg 30 min before pilocarpine injection to block the peripheral effects of pilocarpine. The Li‐PC mice were administered 300 mg/kg pilocarpine (Sigma, P6503) via intraperitoneal injection, which was followed by additional half‐dose treatments every 30 min until the onset of phase four or five seizures. (3) Kainic Acid (KA) Model: Mice in the KA group were stereotaxically injected with kainic acid (0.2 µg in 1 µL saline) into the lateral ventricle (X: 3.1 mm, Y: 2.7 mm, Z: 3.0 mm) under tribromoethanol anesthesia.

Control groups for all models received an equal volume of saline instead of the convulsant agents. Following each injection, all mice were individually observed for 60 min, and seizure severity was scored according to a modified Racine scale: 0, no response; 1, facial myoclonus; 2, head nodding; 3, forelimb clonus; 4, rearing with forelimb clonus; 5, rearing, falling, and severe clonus. Mice exhibiting stage 4 or 5 seizures on three consecutive days were considered fully kindled. Upon completion of the kindling protocol, mice were deeply anesthetized with pentobarbital (60 mg/kg, i.p.). Brain samples were rapidly collected and stored at –80°C for subsequent RT‐qPCR analysis.

### 2.5 Reverse Transcription Real‐Time Quantitative Polymerase Chain Reaction (RT‐qPCR)

To isolate RNA from mouse brain tissue samples, the RNAiso plus reagent (Takara, Dalian, China) was employed. The extracted RNA was subsequently reverse‐transcribed into complementary DNA (cDNA) using the Veriti‐Well Thermal Cycler (Thermo, Wilmington, USA) and the PrimeScript RT reagent Kit with a genomic DNA eraser (Vazyme, Nanjing, China), following the manufacturer's instructions. Specifically, a reaction mixture was prepared containing 1 µg of total RNA, 4 µL of 4× gDNA Wiper Mix, and RNase‐free water, which was incubated at 42°C for 2 min. Then, 4 µL of 5× HiScript II qRT SuperMIX II was added to the mixture, and the reaction was performed at 50°C for 15 min, followed by a brief step at 85°C for 5 s. For the RT‐qPCR reaction, a mixture was prepared consisting of 1 µL of cDNA, 0.4 µL of each forward and reverse primer, 8.2 µL of DEPC‐treated water, and 10 µL of 2× ChamQ qPCR Master Mix (Vazyme, Nanjing, China). The qPCR conditions were as follows: initial denaturation at 95°C for 30 s, followed by 40 cycles of 95°C for 10 s and 60°C for 30 s, with a final extension at 95°C for 15 s. Melting curve analysis was then conducted, ranging from 65.0 to 95.0°C with increments of 0.5°C for 5 s per step. The relative gene expression levels were quantified using the 2^−ΔΔCt^ method (Verbanck et al. 2018). The primer sequences are listed in Table 1, with ACTB serving as the endogenous control for data normalization.

**TABLE 1:** Primers sets used for qPCR.

gene	Forward primer (5’–3’)	Reverse primer (5’–3’)
IER3	CCAGCTACCAACCGAGGAACC	GGCAGAAGATGATGGCGAACAG
TNF	ACGTGGAACTGGCAGAAGAGG	TGAGAAGAGGCTGAGACATAGGC
GPANK1	CCCAGCCCTCCAACCTTCC	CCAGCCACCTCTCAGCAGTAG
ATF6B	AACCTGCCTCTCCGTCTTCTTC	TGTCTTCACTTCCAGAACCTCCTC

### 2.6 Statistical Analysis

Data from RT‐qPCR are presented as mean ± standard error (SEM). For comparisons between two groups, an unpaired Student’ s *t*‐test was used. GraphPad Prism 10 (GraphPad software, La Jolla, USA) was used for data analysis and graph drawing. All experiments were repeated more than 3 times, and *p* < 0.05 was considered statistically significant.

## 3 Results

### 3.1 Polymyositis Was Causally Associated With an Increased Risk of Epilepsy

This MR study investigated the causal relationship between PM/DM and the risk of epilepsy. In the forward MR analysis, the genetic instrument for PM consisted of 7 independent and significant SNPs, whereas the genetic instrument for DM comprised 4 independent and significant SNPs (Table S1). None of the SNPs were excluded by the HEIDI‐outlier test, Steiger filtering, or MR‐PRESSO analysis, indicating robust instrumental variable selection. The analysis employed multiple MR methods, including MR–Egger, weighted median, IVW, simple mode, weighted mode, GSMR, and MR‐PRESSO, to ensure robust and reliable results. The study found significant associations between polymyositis (as exposure) and epilepsy. The IVW analysis revealed a causal association between polymyositis and epilepsy, with an odds ratio (OR) of 1.0302 (95% confidence interval [CI] = 1.0101–1.0507, *p* = 0.0031, below Bonferroni correction threshold of 0.0125). The GSMR analysis (OR = 1.0290, 95 % CI = 1.0081–1.0503, *p* = 0.0063, below Bonferroni correction threshold of 0.0125) also supported the causal association, further demonstrating the reliability of the findings (Figures S1, S2, and 2A). Furthermore, results from the online calculator (https://sb452.shinyapps.io/overlap/) indicate that even under the extreme scenario of 100% sample overlap, the estimated Type 1 error rate remained below 0.05 when using the specified value of the concentration parameter, and below 0.07 when using the conservative value, these results indicate that our MR estimates were not substantially biased by sample overlap (Table S2). These results indicate a potential causal relationship between PM and an increased risk of epilepsy. However, no significant associations were observed between DM and epilepsy across all MR methods.

**FIGURE 2:**
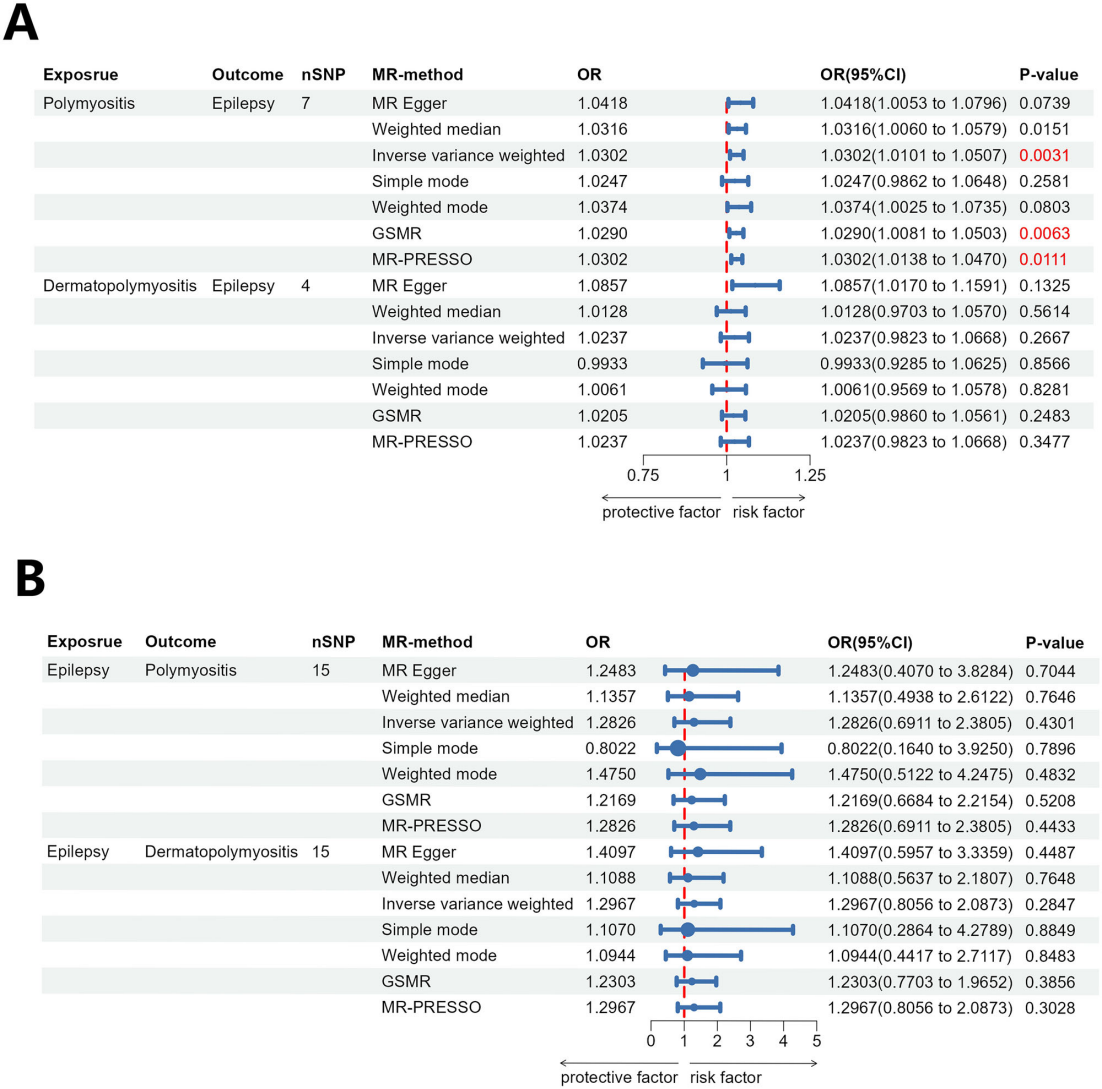
Results of Mendelian randomization (MR) analysis. (A) Forest plot of MR analysis with PM/DM as exposures and epilepsy as the outcome (7 SNPs for PM, 4 SNPs for DM). MR methods include MR–Egger: detects horizontal pleiotropy while estimating causality. Weighted median: robust to partial invalid instrumental SNPs. Inverse variance‐weighted (IVW): assumes all SNPs are valid (as primary analytical approach). Simple/weighted mode: relies on the mode of instrumental effect sizes. GSMR: provides enhanced causal inference by minimizing bias due to correlated pleiotropy (as further validation). MR‐PRESSO: detects and corrects for pleiotropic outliers. Key results: PM shows a significant causal risk for epilepsy via IVW, GSMR, and MR‐PRESSO (*p* < 0.0125 after multiple testing correction, highlighted in red); dermatomyositis has no significant effects. (B) Forest plot of MR analysis with epilepsy as the exposure and PM/DM as outcomes (15 SNPs for each). Using the same MR methods as A, all *p*‐values exceed 0.0125 (no red highlighting), indicating no causal impact of epilepsy on PM/DM. OR (*x*‐axis): <1 = protective factor, >1 = risk factor; blue horizontal lines = 95% CI; red vertical line = null effect (OR = 1). *p*‐Values with statistical significance (typically *p* < 0.0125) are highlighted in red. The application of multiple MR methods with varying assumptions strengthens the robustness of the conclusions, indicating a significant causal role for PM in increasing epilepsy risk, but not for DM.

The heterogeneity and horizontal pleiotropy tests were conducted to validate the Mendelian randomization (MR) analyses. The heterogeneity test results indicated that there was no significant heterogeneity in the MR analyses for polymyositis (PM) or dermatomyositis (DM) as exposures in relation to epilepsy. This was evidenced by non‐significant *Q* statistics across different methods, with all *p*‐values exceeding 0.05 (Table 2). Furthermore, the MR–Egger regression intercept tests for horizontal pleiotropy showed no evidence of pleiotropy, as all intercepts were non‐significant, with *p*‐values greater than 0.05 (Table 3). These results suggest that the genetic instruments utilized are consistent and do not exert influence on the outcomes through alternative pathways, thereby supporting the robustness and reliability of the causal estimates derived from the analyses.

**TABLE 2:** Heterogeneity test of Mendelian randomization analysis results.

Exposure	Outcome	Method	*Q*	*Q*_df	*Q*_pval
Polymyositis	Epilepsy	MR Egger	3.4710	5	0.6278
		IVW	4.0153	6	0.6746
Dermatomyositis	Epilepsy	MR Egger	0.4414	2	0.8020
		IVW	4.6370	3	0.2004
Epilepsy	Polymyositis	MR Egger	16.3120	13	0.2327
		IVW	16.3162	14	0.2944
Epilepsy	Dermatomyositis	MR Egger	16.1116	13	0.2432
		IVW	16.1781	14	0.3026

**TABLE 3:** Pleiotropy test of Mendelian randomization analysis results.

Exposure	Outcome	Egger intercept	SE	*p*‐Value	MR‐PRESSO *p* value
Polymyositis	Epilepsy	−0.0164	0.0222	0.4938	0.7080
Dermatomyositis	Epilepsy	−0.0739	0.0361	0.1771	0.275
Epilepsy	Polymyositis	0.0077	0.1337	0.9547	0.249
Epilepsy	Dermatomyositis	−0.0238	0.1027	0.8204	0.241

### 3.2 Reverse Mendelian Randomization of Epilepsy on Polymyositis

In the reverse MR analysis, we explored the causal effect of epilepsy on the risk of PM/DM. The genetic instrument for epilepsy consisted of 15 SNPs (Table S1). None of these SNPs were removed following HEIDI‐outlier, Steiger‐filtering, or MR‐PRESSO tests, supporting the validity of the genetic instrument. We employed MR–Egger, weighted median, IVW, simple mode, weighted mode methods, GSMR and MR‐PRESSO to examine the causal effects between epilepsy and PM/DM. However, no significant causal association was observed (*p* > 0.0125) (Figures S3, S4, and 2B).

The heterogeneity test results for epilepsy with PM/DM were *p*‐values > 0.05, indicating no significant heterogeneity and suggesting unbiased results (Table 2). Similarly, the MR Egger test for horizontal pleiotropy revealed no clear evidence of horizontal pleiotropy between epilepsy and PM/DM, with *p*‐values>0.05 (Table 3). Sensitivity analysis using the leave‐one‐out approach demonstrated that no individual SNP significantly influenced the study results. Therefore, the bidirectional Two‐sample MR correlation analysis results are deemed stable and reliable.

### 3.3 SNP‐Related Genes and Functions

**FIGURE 3:**
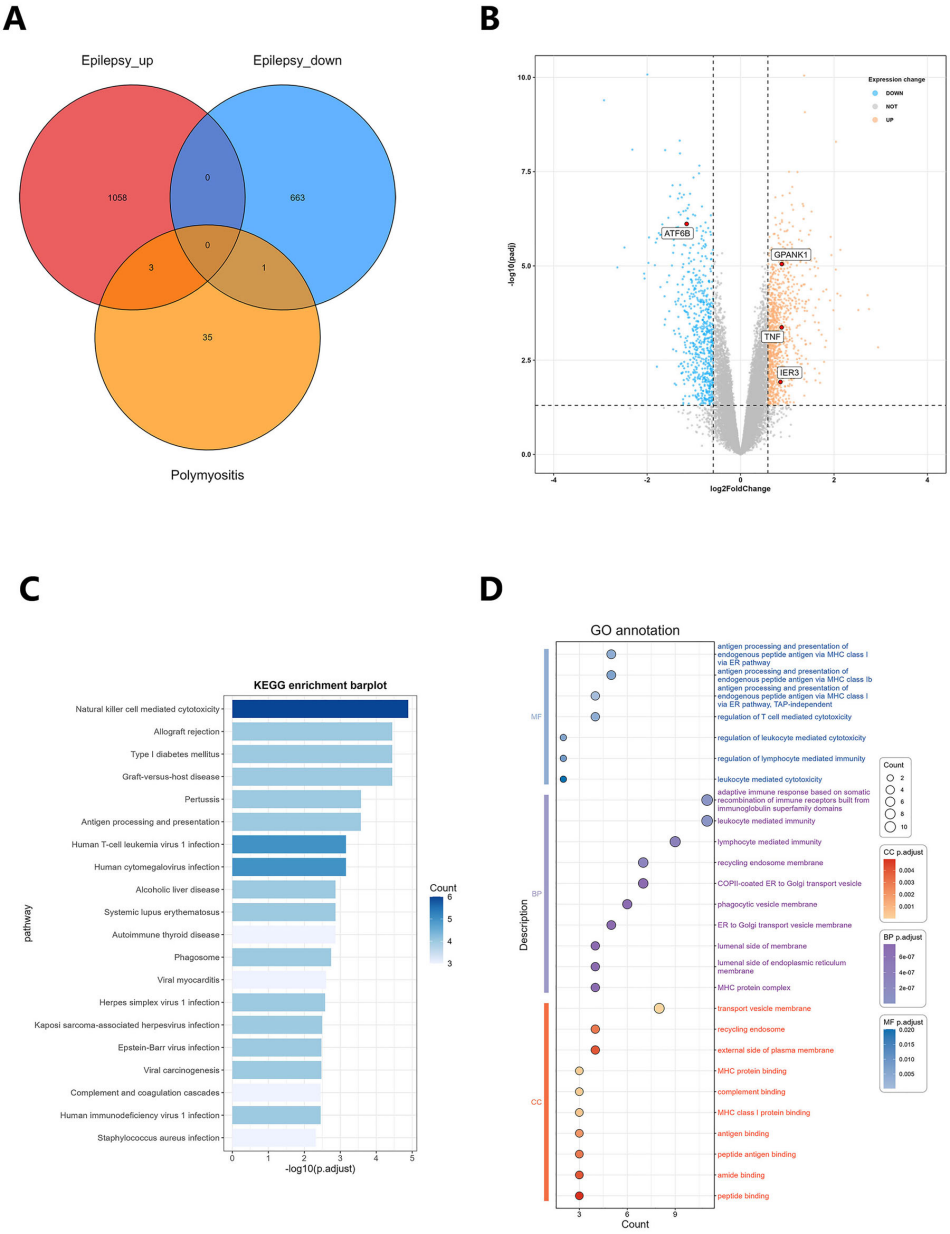
Identification of different expression of SNPs‐related genes, KEGG analysis, and GO analysis. (A) Four DE‐SRGs (IER3, TNF, GPANK1, ATF6B) were obtained by taking the intersections of the SRGs and DEGs. (B) Based on GSE202101 dataset, differential expression analysis was conducted. Orange genes represent significantly high expression in epilepsy, blue genes represent significantly high expression in the control group, and gray genes indicate insignificant changes. (C) KEGG analysis of the SRGs. (D) GO analysis of the SRGs.

**FIGURE 4:**
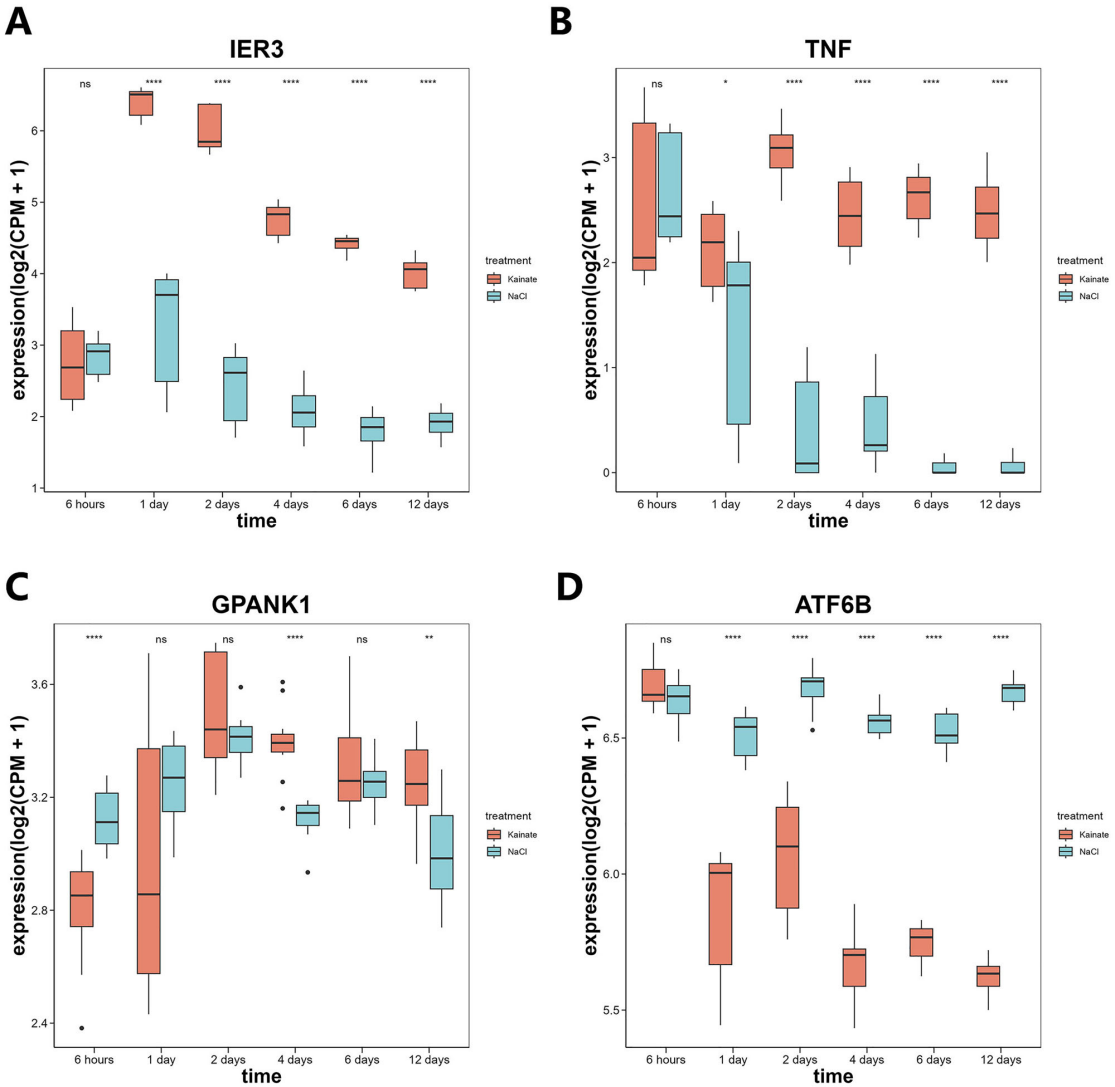
Expression patterns of DE‐SRGs in the CA1 region of the mouse hippocampus at specific time points post‐administration of kainic acid (KA) or NaCl (based on GSE99577 dataset). (A) IER3. (B) TNF. (C) GPANK1. (D) ATF6B. Time points include 6 h, 1 day, 2 days, 4 days, 6 days, and 12 days (****p < 0.0001, ***p < 0.001, **p < 0.01, *p < 0.05, ns indicates not significant).

**FIGURE 5:**
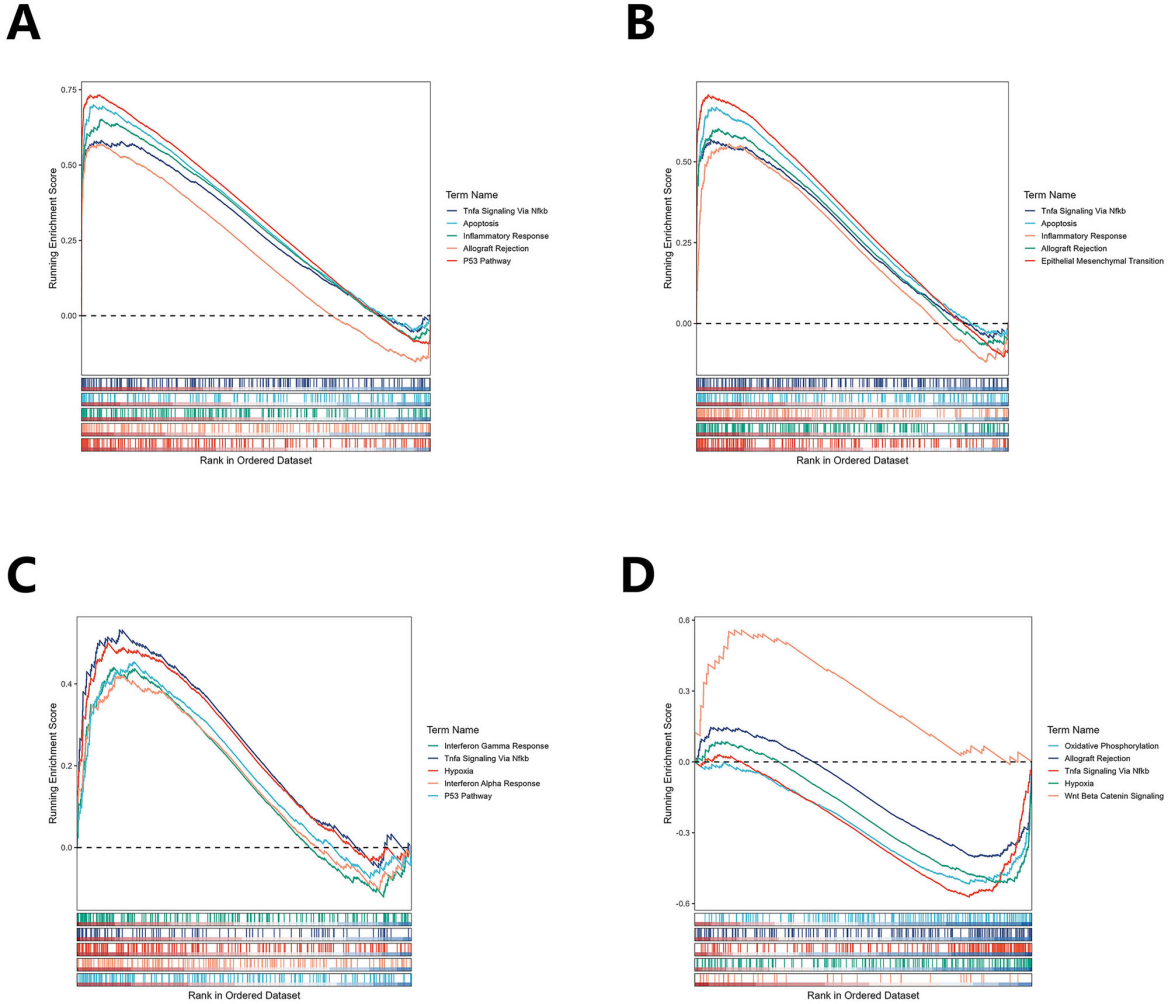
GSEA revealed the enriched pathways of DE‐SRGs (based on GSE63808 dataset). (A) IER3. (B) TNF. (C) GPANK1. (D) ATF6B.

Using the eQTLGen database, we mapped 7 candidate cis‐SNPs, among which only rs2596500 exhibited significant eQTL associations with a total of 39 genes (FDR < 0.05), while no eQTL signals were detected for the remaining 6 variants. The full list of these 39 genes, each associated with rs2596500, is provided in Table S4. We mapped seven SNPs to GTEx hippocampal eQTLs but retrieved no genes, a negative result that most likely reflects the limited sample size of GTEx brain tissue. We therefore retained the 39 genes imputed from eQTLGen as our SRGs. Additionally, the 39 SRGs were enriched in 157 GO items (such as lymphocyte‐mediated immunity, leukocyte‐mediated immunity, and microglial cell activation) and 26 KEGG pathways (such as natural killer cell–mediated cytotoxicity, Allograft rejection, and Type I diabetes mellitus (Figure 3C, D; Tables S5 and S6). Four DE‐SRGs were differentially expressed between the control and epilepsy groups in GSE202101 dataset, including immediate early response 3 (IER3), tumor necrosis factor (TNF), G‐patch domain and ankyrin repeats 1 (GPANK1) (all upregulated), and activating transcription factor 6 beta (ATF6B) (downregulated, Figure 3A, B). Concurrently, our analysis of the GSE99577 dataset elucidated the temporal dynamics of gene expression in response to kainic acid (KA) administration. Specifically, the expression of IER3 exhibited a pronounced peak at 24 h post‐KA injection, followed by a gradual decline over time, yet consistently maintained a higher expression level compared to the control cohort. In contrast, the TNF gene demonstrated sustained elevated expression following KA injection, remaining significantly higher than that observed in the control group one day post‐injection. The expression profile of the GPANK1 was notably more intricate, manifesting an initial upregulation followed by a subsequent downregulation post‐modeling, with levels surpassing those of the control group at the 12‐day mark. Conversely, the ATF6B showed a decline in expression, falling below control group levels after the initial day of modeling (Figure 4). With respect to the single‐gene GSEA of the four DE‐SRGs, IER3 was enriched in 21 pathways, including tumor necrosis factor alpha (TNFα) signaling via nuclear factor kappa‐light‐chain‐enhancer of activated B cells (NF‐κb), apoptosis, inflammatory response. TNF was involved in 20 pathways, such as TNFα signaling via NF‐κb, inflammatory response, and epithelial mesenchymal transition. GPANK1 acted on 14 pathways, for instance, interferon gamma response, TNFα signaling via NF‐κb, and hypoxia. ATF6B played a role in 19 pathways, including oxidative phosphorylation, allograft rejection, and TNFα signaling via NF‐κb (Figure 5; Table S7).

### 3.4 Four DE‐SRG With Immune‐Infiltrating Epilepsy Immune Infiltrating Cells

**FIGURE 6:**
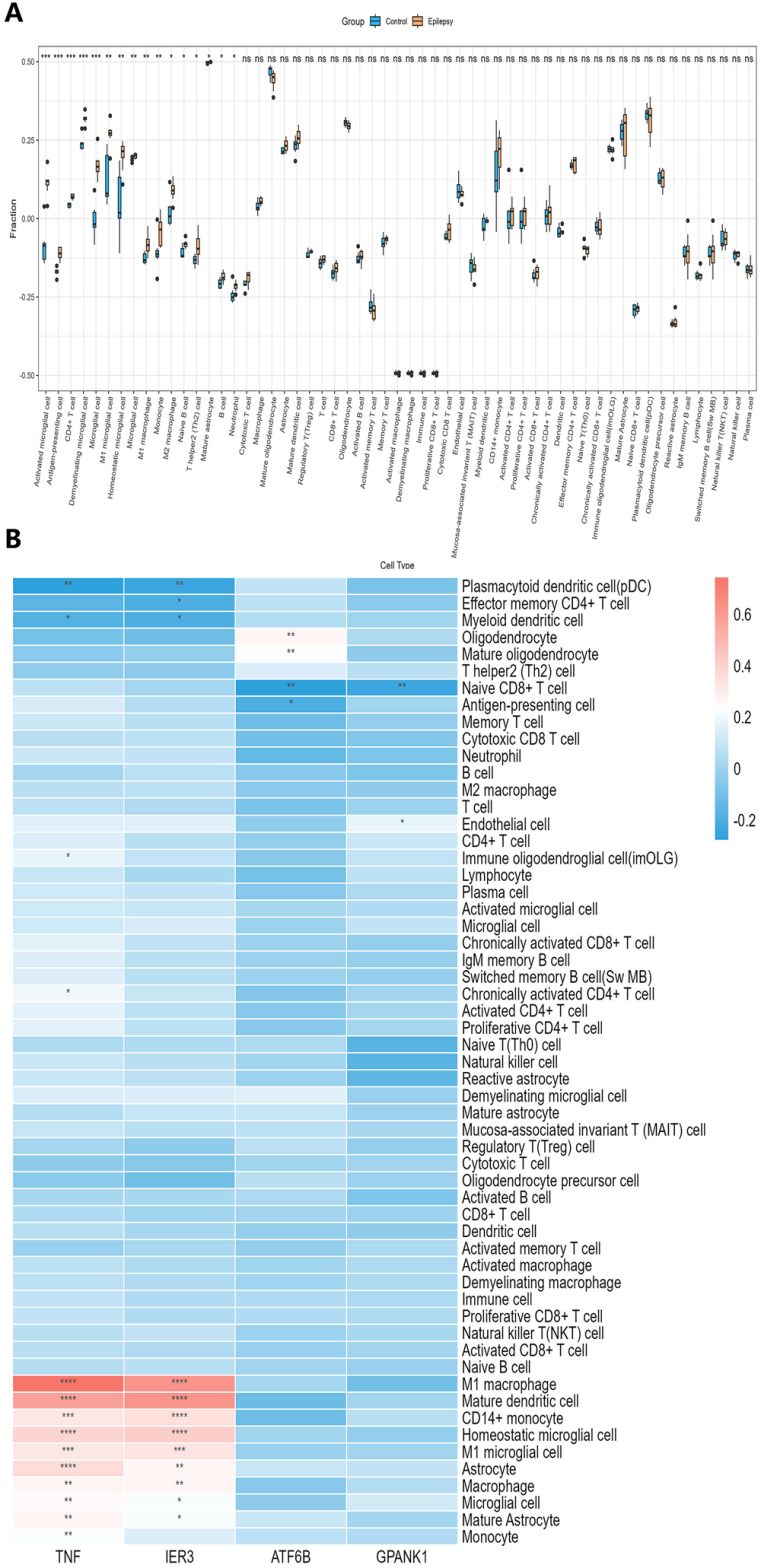
Immune infiltration cells in epilepsy. (A) Boxplot of differences in immune cell infiltration based on ssGSEA algorithm (based on GSE202101 dataset). (B) Correlation of immune cells with 4 DE‐SRGs (based on GSE63808 dataset) (****p < 0.0001, ***p < 0.001, **p < 0.01, *p < 0.05, ns indicates not significant).

Based on the ssGSEA algorithm, we compared 58 immune cell differences between the normal and epilepsy groups in GSE202101. In epilepsy patients, Activated microglial cell, Antigen‐presenting cell, CD4+ T cell, demyelinating microglial cell, M1 microglial cell, M1 macrophage, naive B cell and cytotoxic T cell were upregulated (Figure 6A). Furthermore, we found that TNF and IER3 genes are positively correlated with most immune cell infiltration (*p* < 0.05) (Figure 6B). These results suggest that the DE‐SRGs we identified greatly influence immune cell.

### 3.5 Single‐Cell RNA Sequencing Analysis

**FIGURE 7:**
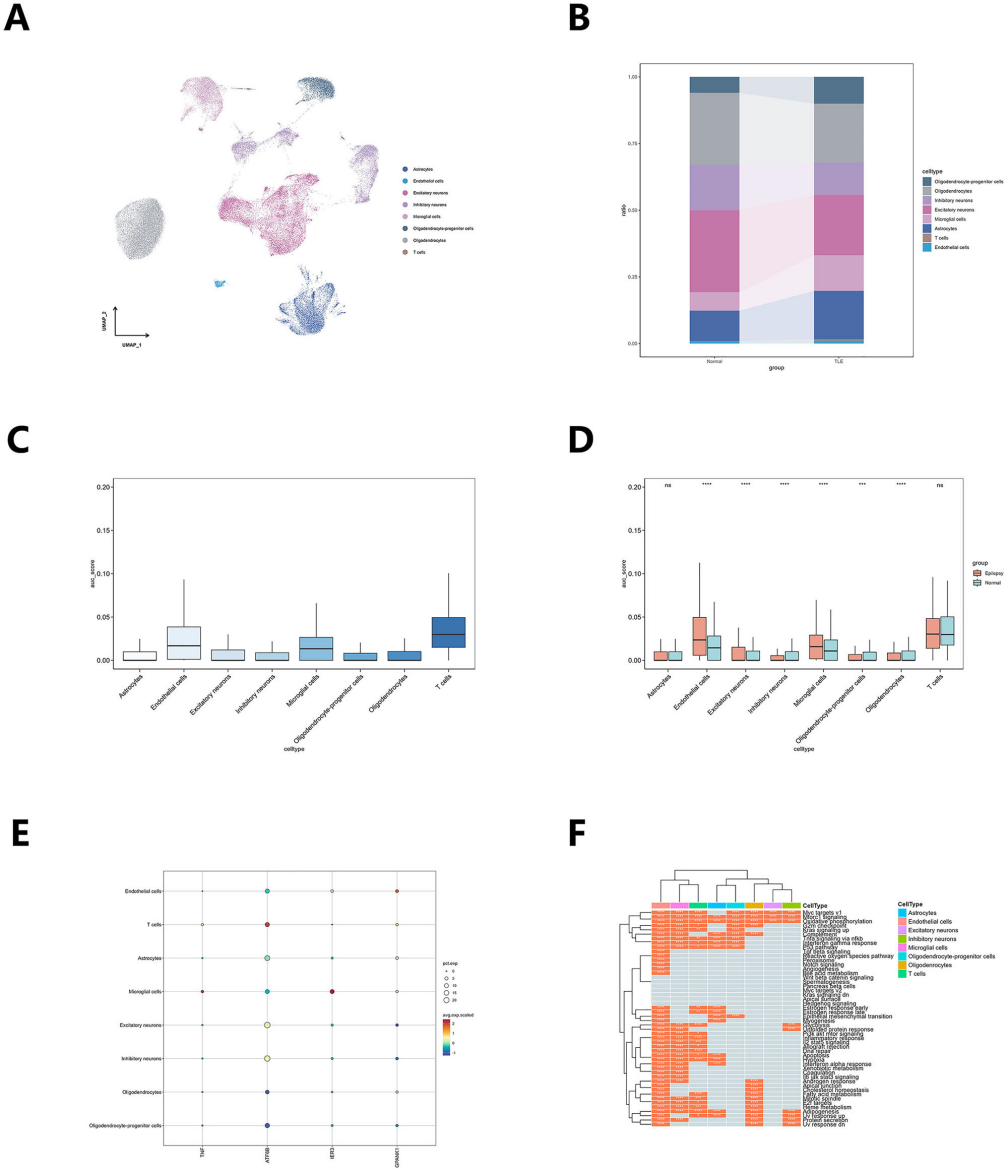
Single‐cell transcriptomics analysis of hippocampal tissues in epilepsy and non‐epilepsy patients (based on GSE190452 dataset). (A) UMAP plot of cell clusters. (B) Bar plot comparing the proportions of each cell type in hippocampal tissues of non‐epilepsy and epilepsy. (C) AUC of SRGs calculated by the AUCell function comparison box plot for various types of cells. (D) AUC of SRGs calculated by the AUCell function comparison box plot for each cell type in hippocampal tissues of non‐epilepsy and epilepsy. (E) The cellular localization patterns of DE‐SRGs. (F) Significant pathways upregulated (orange) in various clusters, including astrocytes, endothelial cells, excitatory neurons, inhibitory neurons, microglial cells, oligodendrocyte‐progenitor cells, oligodendrocytes, T cells (****p < 0.0001, ***p < 0.001, **p < 0.01, *p < 0.05, ns indicates not significant).

Following quality control, the GSE190452 dataset yielded a total of 33,018 genes and 60,029 cells for subsequent analysis. Briefly, cells were sequentially filtered: (i) 3079 cells with nFeature_RNA < 300 or nFeature_RNA > 7000 were removed, leaving 66,887 cells; (ii) 5408 cells with nCount_RNA ≤ 1000 or above the 97th percentile were excluded, leaving 61,479 cells; and (iii) 1450 cells with mitochondrial reads > 10 % were further discarded, resulting in the final 60,029 high‐quality cells. The top 20 highly variable genes were highlighted in a scatter plot (Figure S6A, B). Dimensionality reduction analysis categorized all cells in the GSE190452 dataset into 31 clusters (Figure S6C; Table S8). These clusters were annotated into eight distinct cell types: astrocytes, endothelial cells, excitatory neurons, inhibitory neurons, microglial cells, oligodendrocyte‐progenitor cells, oligodendrocytes, and T cells (Figure 7A). The expression levels of marker genes were deemed appropriate (Figure S6D). Table S9 detailed the number of cells in each cell type. To explore the expression characteristics of SRGs in hippocampal cells affected by epilepsy, the “AUCell” R package was utilized to evaluate the activity of SRGs within each cell type. Cells with higher gene expression exhibited greater AUC values, predominantly in endothelial cells, microglial cells, and T cells (Figure 7C). Notably, AUC values in hippocampal cells of epilepsy patients were generally elevated compared to normal cells, particularly in microglia and endothelial cells (Figure 7D). The study also revealed that TNF and IER3 expression was mainly localized to microglial cells, GPANK1 expression was primarily found in endothelial cells, and ATF6B expression was largely confined to T cells (Figure 7E). To further investigate the functional differences between epileptic and normal hippocampal cells, this study compared the upregulation and downregulation of pathways in patients with and without epileptic seizures. Endothelial cells, microglia, and T cells exhibited high consistency in pathway activation, with pathways such as inflammatory response, allograft rejection, and Myc targets v1 being highly upregulated (Figure 7F).

### 3.6 IER3, TNF, GPANK1, and ATF6B mRNA Expression Is Increased in the Hippocampal Tissue of PTZ‐Kindled Mouse

**FIGURE 8:**
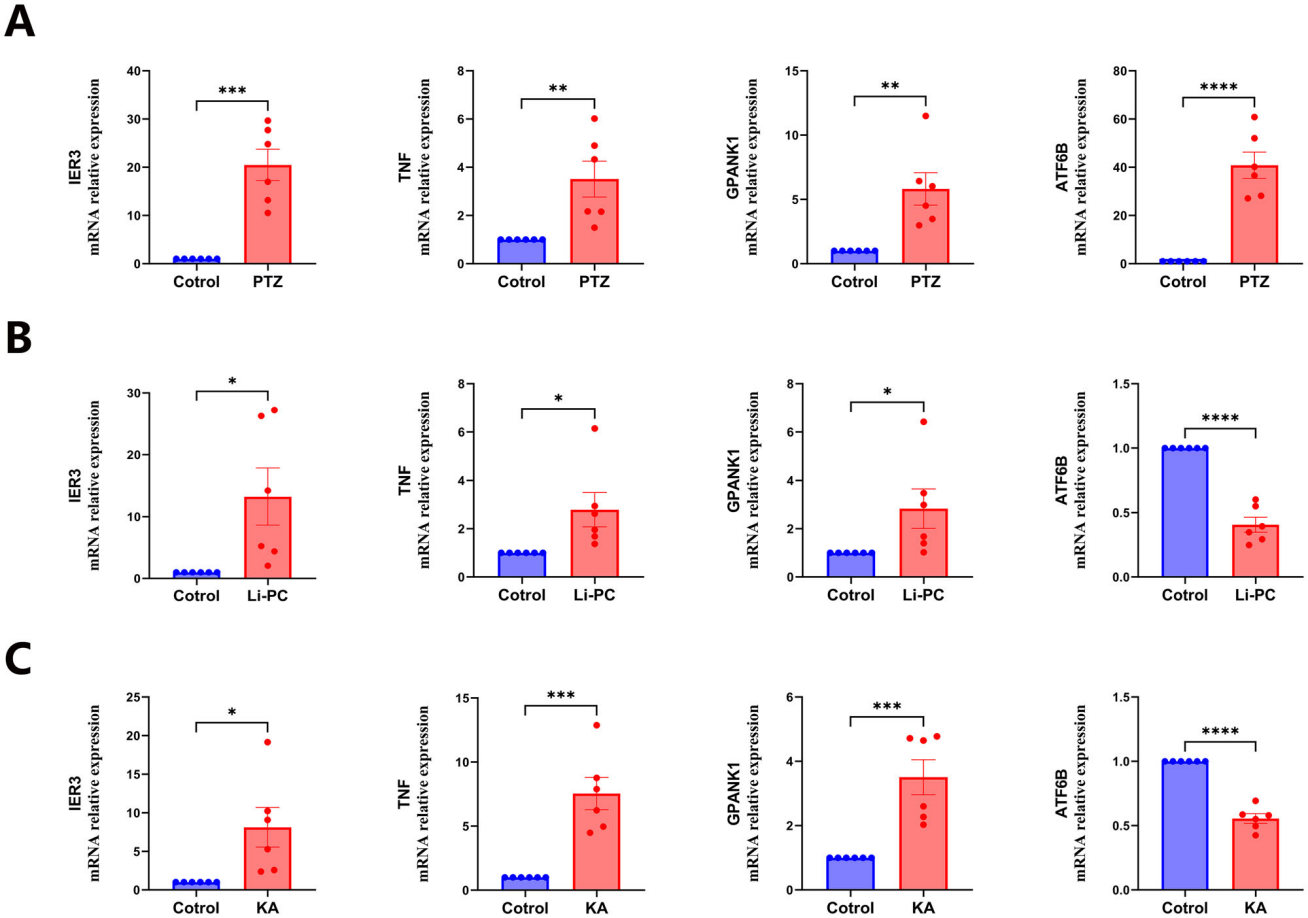
RT‐qPCR of IER3, TNF, GPANK1, and ATF6B mRNA in the hippocampal tissue of epilepsy mouse model. (A) RT‐qPCR revealed that the mRNA level of the IER3, TNF, GPANK1, and ATF6B gene was higher in hippocampus of PTZ group mouse than control. (B) RT‐qPCR revealed that the mRNA level of the IER3, TNF, GPANK1 gene was higher in hippocampus of Li‐PC‐kindled mouse than control, while ATF6B mRNA expression was decreased. (C) RT‐qPCR revealed that the mRNA level of the IER3, TNF, GPANK1 gene was higher in hippocampus of KA‐kindled mouse than control, while ATF6B mRNA expression was decreased. (*n* = 6, ****p < 0.0001, ***p < 0.001, **p < 0.01, *p < 0.05, error bar indicates standard error).

RT‐qPCR revealed that the mRNA level of the IER3, TNF, GPANK1, and ATF6B gene was higher in hippocampus of PTZ group mouse than control (Figure 8A).

### 3.7 IER3, TNF, and GPANK1 mRNA Expression Is Increased in the Hippocampal Tissue of Li‐PC‐ and KA‐Kindled Mouse, While ATF6B mRNA Expression Is Decreased

RT‐qPCR revealed that the mRNA level of the IER3, TNF, and GPANK1 gene was higher in hippocampus of Li‐PC (Figure 8B) and KA (Figure 8C) kindled mouse than control, while ATF6B mRNA expression was decreased.

## 4 Discussion

In this study, we took advantage of the two‐sample MR method with multiple methods including IVW and GSMR, to analyze the causal relationship between polymyositis (PM)/dermatomyositis (DM) and epilepsy. The main results of MR analysis consistently suggested that polymyositis was causally associated with a higher risk of epilepsy, while DM was no causally associated with epilepsy. This result is consistent with a recent cross‐sectional study by Ella et al. (Nissan et al. 2021), which reported PM is associated with a higher rate of epilepsy compared to controls. However, our study extends these findings by establishing a causal link, rather than merely an association. The heterogeneity and pleiotropy tests further bolster the validity of our MR analysis. The absence of significant heterogeneity and the non‐significant intercepts in the MR–Egger regression tests suggest that our genetic instruments are not confounded by pleiotropic effects, which is a common concern in observational studies of this nature. Interestingly, our reverse MR analysis, which investigated the causal effect of epilepsy on the risk of PM, did not yield significant results. This discrepancy may be attributed to the complex bidirectional relationship between immune‐mediated diseases and neurological disorders, where the immune system's role in epilepsy might be more pronounced than its susceptibility to the disease itself. In addition, the absence of a DM‐epilepsy link may reflect fundamental differences in immunopathology. PM is characterized by T cell–mediated cytotoxicity, with CD8+ T cell infiltration directly damaging muscle fibers. This adaptive cellular immune response is also implicated in neuroinflammation and epileptogenesis, as T lymphocytes are found in the CNS in various epilepsy syndromes, and systemic T cell–driven disorders (e.g., rheumatoid arthritis) carry an elevated epilepsy risk (Bauer et al. 2017; Steriade et al. 2021). Conversely, DM is defined by a humoral immune response, involving autoantibodies, B cells, and complement‐mediated vascular injury (McHugh and Tansley 2018; Shinjo et al. 2013). While both conditions involve proinflammatory cytokines, the T cell–driven process in PM may more readily promote neuroinflammation and blood–brain barrier disruption, thereby facilitating seizures. These differences highlight that immunotherapeutic strategies targeting T cells might be more relevant for PM‐related epilepsy, whereas B cell depletion is a cornerstone in DM treatment (McHugh and Tansley 2018; Shinjo et al. 2013).

Our study has identified a significant association between PM‐related SNPs and epilepsy, providing a genetic basis for the observed clinical link between these two conditions. The identification of 39 genes associated with IVs from the eQTLGen database, particularly the SNP rs2596500 linked to polymyositis, underscores the genetic complexity of these disorders. The differential expression of four DE‐SRGs between control and epilepsy groups, including IER3, TNF, GPANK1 (upregulated), and ATF6B (downregulated), further elucidates the molecular mechanisms underlying the epilepsy phenotype. Our results are consistent with prior studies that have implicated these genes in immune response and inflammation (Arlt and Schäfer 2011; Firdous et al. 2021; Kumar et al. 2022; Nguyen et al. 2021). To verify the actual expression of these genes in epilepsy, the mRNA levels of IER3, TNF, GPANK1, and ATF6B were detected in the hippocampus of commonly used epilepsy mouse models, including PTZ, Li‐PC and KA models. The results showed that the expression levels of IER3, TNF, GPANK1, and ATF6B in the hippocampus of Li‐PC and KA mice were consistent with the results of transcriptome data. Although the results of ATF6B in the hippocampus of the PTZ model are different from the transcriptome data, we consider that it is related to the differential expression of ATF6B in different drug‐induced epilepsy models. Divergent ATF6B expression in PTZ models may reflect acute seizure induction mechanisms versus chronic neuroinflammation in Li‐PC/KA paradigms, warranting further investigation into temporal dynamics of endoplasmic reticulum stress responses. Therefore, we will use Li‐PC and KA models in subsequent experiments to further verify the specific molecular mechanism.

The enrichment of the 39 SRGs in 157 GO terms and 26 KEGG pathways points to the involvement of these genes in critical biological processes. The significant enrichment in immune‐related GO terms, such as lymphocyte‐ and leukocyte‐mediated immunity, and microglial cell activation, supports the notion that immune dysregulation is a central feature of epilepsy. Similarly, the enrichment in Kyoto Encyclopedia of Genes and Genomes (KEGG) pathways like natural killer cell–mediated cytotoxicity and allograft rejection further emphasizes the immune‐mediated nature of the epilepsy pathogenesis (Vezzani et al. 2022; Sanz and Garcia‐Gimeno 2020). The single‐gene GSEA of the four DE‐SRGs revealed that IER3, TNF, GPANK1, and ATF6B are involved in numerous pathways associated with inflammation, apoptosis, and immune response. The involvement of these genes in TNFα signaling via NF‐κb and other inflammatory pathways is particularly noteworthy, as it suggests a potential therapeutic target for modulating the inflammatory response in epilepsy. This is supported by studies showing that inhibition of TNFα signaling can ameliorate seizure activity (Henning et al. 2023). The identification of these DE‐SRGs and their associated pathways provides a foundation for future research into the molecular mechanisms linking polymyositis and epilepsy. Further investigation into the functional roles of these genes, particularly in the context of immune cell infiltration and activation, is warranted. Understanding the precise mechanisms by which these genes contribute to epilepsy may lead to the development of novel therapeutic strategies targeting the immune system. What's more, the dysregulation of TNF and IER3 pathways in PM‐associated epilepsy suggests therapeutic potential for targeted immunomodulation (e.g., TNF signaling blockade), which may mitigate seizure susceptibility in high‐risk patients.

The study found that epilepsy patients have higher immune scores than controls, and there are significant differences in the levels of immune cell infiltration. Sixteen key immune cells associated with epileptogenesis were identified by the ssGSEA algorithm. The upregulation of these immune cells underscores the complexity of the immune response in epilepsy and indicates a potential shift towards a proinflammatory state. This is consistent with the neuroinflammatory hypothesis of epilepsy, which posits that inflammation contributes to seizure susceptibility and progression (Sinha et al. 2021). Our results expand on this hypothesis by providing a detailed molecular landscape of immune cell infiltration in epilepsy. Moreover, our study identified a positive correlation between the expression of TNF and IER3 genes and the infiltration of most immune cells. This correlation is significant as it links the genetic makeup of epilepsy patients to the immunological changes observed in the disease state. TNF, a key proinflammatory cytokine, has been previously implicated in epilepsy, with studies showing its increased expression in the hippocampus of patients with temporal lobe epilepsy (Huang et al. 2022). IER3, an immediate early response gene, is known for its role in the regulation of immune and inflammatory responses, but its role in epilepsy has not been reported, which deserves further study. The correlation between DE‐SRGs and immune cell infiltration provides a molecular link between genetic susceptibility and immune‐mediated inflammation in epilepsy. This is in line with numerous studies that have highlighted the role of genetic factors in shaping the immune response in neurological disorders. Our findings add to the body of evidence suggesting that targeting immune pathways, particularly those involving TNF and IER3, may offer therapeutic benefits in epilepsy management.

Our single‐cell RNA sequencing analysis of the GSE190452 dataset has provided a comprehensive view of the cellular landscape in epilepsy, revealing significant heterogeneity and specific cellular changes associated with the disease. The use of the “AUCell” R package to assess the activity of SNP‐related genes (SRGs) in each cell line revealed higher AUC values in endothelial cells, microglial cells, and T cells, indicating increased gene expression in these cell types. This is in line with prior research suggesting that these cells play a crucial role in neuroinflammatory processes associated with epilepsy. Our finding that AUC values in hippocampal cells of epilepsy patients are generally higher than in normal individuals, particularly in microglia and endothelial cells, further supports the notion that these cells contribute to the epileptic phenotype. The comparison of pathway activation in epileptic and non‐epileptic hippocampal cells revealed significant upregulation of pathways related to inflammatory response, allograft rejection, and Myc targets v1 in endothelial cells, microglia, and T cells. This is consistent with previous work that has implicated these pathways in the immune response and epilepsy. This finding is novel and suggests that crosstalk between the immune system and the brain may be a key factor in epilepsy pathogenesis, building on studies that have shown immune cell infiltration in the epileptic brain. The enhanced pathway activation we observed in endothelial cells, microglia, and T cells suggest that these cells may be key mediators of the immune response in epilepsy. This aligns with the growing body of evidence that implicates immune activation in the development and progression of epilepsy. Our findings may also have therapeutic implications, as targeting these specific cell types or their associated pathways could potentially offer a new avenue for treatment.

The strengths of this study lie in its multifaceted approach, integrating bioinformatics analysis and Mendelian randomization (MR) methods to thoroughly examine the relationship between polymyositis (PM) and dermatomyositis (DM) with epilepsy from diverse angles. This comprehensive methodology enhances the reliability and comprehensiveness of the findings. Moreover, the study leveraged publicly accessible databases and extensive gene expression datasets, providing a robust foundation for the research. Additionally, rigorous statistical analysis was employed to ensure the accuracy and reliability of the results.

Despite these strengths, the study has several notable limitations. First, we did not investigate potential heterogeneity in the causal relationship between polymyositis (PM) and epilepsy by conducting stratified Mendelian randomization (MR) analyses across clinically relevant subgroups, such as epilepsy subtype, disease duration, and medication status. This unmeasured clinical heterogeneity may mask subgroup‐specific effects and limit the generalizability of our findings. For example, if the causal effect of PM exposure is restricted to certain epilepsy subtypes or stages of disease progression, our overall estimate may represent a diluted or null average effect. Future work in stratified populations is warranted to further investigate the heterogeneity of the effect of PM on epilepsy in specific sub‐populations (Cosia et al. 2022), which could be a basis of clinical studies. Second, due to the limited number of available SNPs, we used a relaxed significance threshold (*p* < 5 × 10^−6^) for instrument selection, which may increase the risk of weak instrument bias. Although we applied *F*‐statistics and sensitivity analyses to assess instrument strength and pleiotropy, residual bias cannot be entirely excluded. Third, GWAS summary statistics are susceptible to the winner's curse, potentially leading to overestimated genetic effects and biased causal estimates (Burgess et al. 2013). Fourth, although we took steps to validate instrumental variables, further MR designs robust to invalid instruments are warranted. Fifth, this study relied on GWAS summary statistics, with FinnGen data included in both the exposure (PM/DM) and outcome (epilepsy) datasets, resulting in sample overlap that may potentially bias causal estimates. We evaluated this risk using an online tool (https://sb452.shinyapps.io/overlap/), which indicated that our Mendelian randomization estimates were not substantially biased by the presence of sample overlap. Meanwhile, missing data primarily concerned uncaptured clinical covariates, such as polymyositis disease activity, epilepsy seizure frequency, and comorbid autoimmune conditions. This limitation introduces unmeasured confounding, which may obscure heterogeneous treatment effects—for instance, a stronger causal effect for severe versus mild polymyositis. To address these limitations and strengthen the conclusions, future research should prioritize the following directions: (1) Employ individual‐level data Mendelian randomization analyses to directly exclude overlapping participants, adjust for detailed confounders (e.g., age, sex, comorbidities), and enable precise stratified analyses. (2) Collect comprehensive clinical information to minimize missing data and better capture disease heterogeneity. (3) Validate findings using complementary experimental designs, such as longitudinal cohort studies, genome‐wide association studies in diverse populations, or functional experiments to explore underlying mechanisms. Finally, although we validated the altered expression of IER3, TNF, GPANK1, and ATF6B in multiple epilepsy models, we did not investigate the correlation between their expression levels and behavioral seizure outcomes (e.g., Racine scores, seizure frequency). This limits our ability to directly associate these molecular changes with functional phenotypes. Genetic intervention experiments (e.g., knockout or overexpression of candidate genes) were not performed, which would have provided stronger evidence for their functional roles in epileptogenesis. These aspects will be addressed in our future studies, where we plan to systematically examine the relationship between molecular expression and behavioral manifestations, as well as conduct functional validation experiments.

## 5 Conclusion

Our investigation offers compelling evidence supporting a causal link between polymyositis (PM) and epilepsy, underscoring the significance of immune‐mediated mechanisms in epilepsy's pathogenesis. No such association was found for dermatomyositis (DM). Through advanced bioinformatics and statistical analyses, this study meticulously dissects the intricate relationship between PM and epilepsy, pinpointing pivotal genes and pathways. Notably, genes such as IER3, TNF, GPANK1, and ATF6B emerge as key players. However, it is crucial to acknowledge that these findings necessitate further validation, given the inherent limitations in data selection and analysis techniques. Overall, this study enriches our understanding of epilepsy, advocating for targeted molecular and immunological strategies in future therapeutic and research endeavors.

## Author Contributions

**Ying Liu**: data curation; validation; formal analysis; funding acquisition; writing – original draft; writing – review & editing. **Yuhang Yu**: methodology; software; visualization; resources; writing – original draft; writing – review & editing. **Chulong Fang**: data curation; validation; formal analysis. **Qingzhong Wu**: validation; formal analysis. **Lingli Ou**: validation; formal analysis. **Jianhao Xiao**: validation; formal analysis. **Kui Duan**: formal analysis. **Ting Jiang**: formal analysis. **Lan Ye**: conceptualization; investigation; supervision; funding acquisition; writing – review & editing. **Xiao Hu**: conceptualization; investigation; writing – review & editing. **Zhanhui Feng**: conceptualization; investigation; supervision; funding acquisition; project administration; writing – review & editing.

## Funding

The authors declare financial support was received for the research, authorship, and publication of this article. This study was supported by a grant from the National Natural Science Foundation of China (Grant No. 82360266); Guizhou Provincial Basic Research Program (Grant No. Qiankehe basic‐ZK [2023] general 395; Qiankehe basic‐ZK [2023] general 324; Qiankehe basic‐ZK [2024] general 228; Qiankehe basic‐MS[2025]548); The Cultivate project 2021 for National Natural Science Foundation of China, the Affiliated Hospital of Guizhou Medical University (Grant No. gyfynsfc‐2021‐22); Key Advantageous Discipline Construction Project of Guizhou Provincial Health Commission in 2023, China; Key lab of acute brain injury and function repair in Guizhou Medical University (Grant No. [2024] fy0071); Guizhou Provincial Key Laboratory of Brain Function and Brain Disease Prevention and Treatment (Grant No. [Qiankehe platform ZSYS(2025)030]); Talent Program of Guizhou Provincial People's Hospital (Grant No. [2024]‐44).

## Conflicts of Interest

The authors declare that they have no conflicts of interest.

## Supporting information




**Table S1** The Used SNP data for Mendelian randomization.

**Table S2** The Bias and Type 1 error rate for Mendelian randomization with sample overlap.

**Table S3** 58 immune cell features from CellMarker 2.0 database in immune infiltration analysis.

**Table S4** The SNP‐to‐gene data.

**Table S5** The result of GO Analysis.

**Table S6** The result of KEGG Analysis.

**Table S7** The result of Single‐gene Gene Set Enrichment Analysis.

**Table S8** The Annotation of cell clusters from single‐cell RNA‐seq analysis.

**Table S9** The Distribution of cell counts from single‐cell RNA sequencing analysis.

Figures S1–S6

**Figure S1** The Mendelian Randomization Analysis of Polymyositis (exposure) on Epilepsy.

**Figure S2** The Mendelian Randomization Analysis ofDermatomyositis(exposure) on epilepsy.

**Figure S3** The Mendelian Randomization Analysis ofepilepsy(exposure) on Polymyositis.

**Figure S4** The Mendelian Randomization Analysis ofepilepsy(exposure) on Dermatomyositis.

**Figure S5** The boxplot illustrates the distribution of gene expression values across samples in the GSE202101 dataset.

**Figure S6** The Single cell RNA‐seq data analysis.

## Data Availability

All data utilized in this study are publicly accessible. Data are also available from the corresponding author upon reasonable request. Generate a chart with the code provided at https://github.com/dreamyuyu/PM‐Epilepsy‐figure.

## References

[brb371148-bib-0001] Amanat, M. , R. D. Thijs , M. Salehi , and J. W. Sander . 2019. “Seizures as a Clinical Manifestation in Somatic Autoimmune Disorders.” Seizure: The Journal of the British Epilepsy Association 64: 59–64. 10.1016/j.seizure. 30562654

[brb371148-bib-0002] Arlt, A. , and H. Schäfer . 2011. “Role of the Immediate Early Response 3 (IER3) Gene in Cellular Stress Response, Inflammation and Tumorigenesis.” European Journal of Cell Biology 90, no. 6‐7: 545–552. 10.1016/j.ejcb.2010.10.002. 21112119

[brb371148-bib-0003] Bauer, J. , A. J. Becker , W. Elyaman , et al. 2017. “Innate and Adaptive Immunity in Human Epilepsies.” Epilepsia 58, no. 3: 57–68. 10.1111/epi.13784. 28675562 PMC5535008

[brb371148-bib-0004] Bowden, J. , G. Hemani , and G. D. Smith . 2018. “Invited Commentary: Detecting Individual and Global Horizontal Pleiotropy in Mendelian Randomization—A Job for the Humble Heterogeneity Statistic?” American Journal of Epidemiology 187, no. 12: 2681–2685. 10.1093/aje/kwy185. 30188969 PMC6269239

[brb371148-bib-0005] Burgess, S. , A. Butterworth , and S. G. Thompson . 2013. “Mendelian Randomization Analysis With Multiple Genetic Variants Using Summarized Data.” Genetic Epidemiology 37, no. 7: 658–665. 10.1002/gepi.21758. 24114802 PMC4377079

[brb371148-bib-0006] Burgess, S. , N. M. Davies , and S. G. Thompson . 2016. “Bias Due to Participant Overlap in Two‐Sample Mendelian Randomization.” Genetic Epidemiology 40, no. 7: 597–608. 10.1002/gepi.21998. 27625185 PMC5082560

[brb371148-bib-0007] Burgess, S. , and S. G. Thompson . 2017. “Interpreting Findings From Mendelian Randomization Using the MR‐Egger Method.” European Journal of Epidemiology 32, no. 5: 377–389. 10.1007/s10654-017-0276-5. 28527048 PMC5506233

[brb371148-bib-0008] Burgess, S. , and S. G. Thompson CRP CHD Genetics Collaboration . 2011. “Avoiding Bias From Weak Instruments in Mendelian Randomization Studies.” International Journal of Epidemiology 40, no. 3: 755–764. 10.1093/ije/dyr036. 21414999

[brb371148-bib-0009] Chali, F. , G. Milior , S. Marty , et al. 2019. “Lipid Markers and Related Transcripts During Excitotoxic Neurodegeneration in kainate‐treated Mice.” European Journal of Neuroscience 50, no. 1: 1759–1778. 10.1111/ejn.14375. 30767299

[brb371148-bib-0010] Chen, J. , H. Chen , Q. Zhu , et al. 2022. “Age at Menarche and Ischemic Heart Disease: An Update Mendelian Randomization Study.” Frontiers in Genetics 13: 942861. 10.3389/fgene.2022.942861. 36406117 PMC9671358

[brb371148-bib-0011] Chen, Z. P. , S. Wang , X. Zhao , et al. 2023. “Lipid‐Accumulated Reactive Astrocytes Promote Disease Progression in Epilepsy.” Nature Neuroscience 26, no. 4: 542–554. 10.1038/s41593-023-00542-4. 36941428

[brb371148-bib-0012] Cosia, C. , D. Gill , R. Benítez , T. Pérez , N. Malats , and S. Burgess . 2022. “Avoiding Collider Bias in Mendelian Randomization When Performing Stratified Analyses.” European Journal of Epidemiology 37, no. 7: 671–682. 10.1007/s10654-022-00879-0. 35639294 PMC9329404

[brb371148-bib-0013] Dalakas, M. C. 2015. “Inflammatory Muscle Diseases.” New England Journal of Medicine 372, no. 18: 1734–1747. 10.1056/NEJMra1402225. 25923553

[brb371148-bib-0014] Davies, N. M. , M. V. Holmes , and G Davey Smith . 2018. “Reading Mendelian Randomisation Studies: A Guide, Glossary, and Checklist for Clinicians.” Bmj 362: k601. 10.1136/bmj.k601. 30002074 PMC6041728

[brb371148-bib-0015] Devinsky, O. , A. Vezzani , T. J. O'Brien , et al. 2018. “Epilepsy.” Nature Reviews Disease primers 4: 18024. 10.1038/nrdp.2018.24. 29722352

[brb371148-bib-0016] Dobrijevic, E. , A. van Zwieten , K. Kiryluk , A. J. Grant , G. Wong , and A. Teixeira‐Pinto . 2023. “Mendelian Randomization for Nephrologists.” Kidney International 104, no. 6: 1113–1123. 10.1016/j.kint.2023.09.016. 37783446

[brb371148-bib-0017] Erlangsen, A. , E. Stenager , Y. Conwell , et al. 2020. “Association Between Neurological Disorders and Death by Suicide in Denmark.” Jama 323, no. 5: 444–454. 10.1001/jama.2019.21834. 32016308 PMC7042859

[brb371148-bib-0018] Fabene, P. F. , G. Navarro Mora , M. Martinello , et al. 2008. “A Role for Leukocyte‐Endothelial Adhesion Mechanisms in Epilepsy.” Nature Medicine 14, no. 12: 1377–1383. 10.1038/nm.1878. PMC2710311 19029985

[brb371148-bib-0019] Firdous, A. , S. Sarwar , F. A. Shah , et al. 2021. “Contribution of Attenuation of TNF‐α and NF‐κB in the Anti‐Epileptic, Anti‐Apoptotic and Neuroprotective Potential of Rosa Webbiana Fruit and Its Chitosan Encapsulation.” Molecules (Basel, Switzerland) 26, no. 8: 2347. 10.3390/molecules26082347. 33920713 PMC8073239

[brb371148-bib-0020] Gu, Y. , Q. Jin , J. Hu , et al. 2023. “Causality of Genetically Determined Metabolites and Metabolic Pathways on Osteoarthritis: A Two‐Sample Mendelian Randomization Study.” Journal of translational medicine 21, no. 1: 357. 10.1186/s12967-023-04165-9. 37259122 PMC10230782

[brb371148-bib-0021] Hänzelmann, S. , R. Castelo , and J. Guinney . 2013. “GSVA: Gene Set Variation Analysis for Microarray and RNA‐seq Data.” BMC Bioinformatics [Electronic Resource] 14: 7. 10.1186/1471-2105-14-7. 23323831 PMC3618321

[brb371148-bib-0022] Hao, Y. , T. Stuart , M. H. Kowalski , et al. 2024. “Dictionary Learning for Integrative, Multimodal and Scalable Single‐Cell Analysis.” Nature Biotechnology 42, no. 2: 293–304. 10.1038/s41587-023-01767-y. PMC10928517 37231261

[brb371148-bib-0023] He, Y. , H. Zhang , L. Ma , et al. 2023. “Identification of TIMP1 as an Inflammatory Biomarker Associated With Temporal Lobe Epilepsy Based on Integrated Bioinformatics and Experimental Analyses.” Journal of Neuroinflammation 20, no. 1: 151. 10.1186/s12974-023-02837-3. 37365625 PMC10294353

[brb371148-bib-0024] Hemani, G. , K. Tilling , and G Davey Smith . 2017. “Orienting the Causal Relationship Between Imprecisely Measured Traits Using GWAS Summary Data.” PLos Genetics 13, no. 11:e1007081. 10.1371/journal.pgen.1007081. 29149188 PMC5711033

[brb371148-bib-0025] Hemani, G. , J. Zheng , B. Elsworth , et al. 2018. “The MR‐Base Platform Supports Systematic Causal Inference Across the Human Phenome.” Elife 7: e34408. 10.7554/eLife.34408. 29846171 PMC5976434

[brb371148-bib-0026] Henning, L. , H. Antony , A. Breuer , et al. 2023. “Reactive Microglia Are the Major Source of Tumor Necrosis Factor Alpha and Contribute to Astrocyte Dysfunction and Acute Seizures in Experimental Temporal Lobe Epilepsy.” Glia 71, no. 2: 168–186. 10.1002/glia.24265. 36373840

[brb371148-bib-0027] Hernández Martínez, A. , R. Madurga , N. García‐Romero , and Á Ayuso‐Sacido . 2022. “Unravelling Glioblastoma Heterogeneity by Means of Single‐Cell RNA Sequencing.” Cancer Letters 527: 66–79. 10.1016/j.canlet.2021.12.008. 34902524

[brb371148-bib-0028] Hildebrandt, M. , K. Amann , R. Schröder , et al. 2008. “White Matter Angiopathy Is Common in Pediatric Patients With Intractable Focal Epilepsies.” Epilepsia 49, no. 5: 804–815. 10.1111/j.1528-1167.2007.01514.x. 18266747

[brb371148-bib-0029] Hu, C. , T. Li , Y. Xu , et al. 2023. “CellMarker 2.0: an Updated Database of Manually Curated Cell Markers in human/Mouse and Web Tools Based on scRNA‐seq Data.” Nucleic Acids Research 51, no. D1: D870–D876. 10.1093/nar/gkac947. 36300619 PMC9825416

[brb371148-bib-0030] Huang, W. Y. , Y. L. Lai , K. H. Liu , et al. 2022. “TNFα‐Mediated Necroptosis in Brain Endothelial Cells as a Potential Mechanism of Increased Seizure Susceptibility in Mice Following Systemic Inflammation.” Journal of Neuroinflammation 19, no. 1: 29. 10.1186/s12974-022-02406-0. 35109859 PMC8809013

[brb371148-bib-0031] Johnson, M. R. , J. Behmoaras , L. Bottolo , et al. 2015. “Systems Genetics Identifies Sestrin 3 as a Regulator of a Proconvulsant Gene Network in Human Epileptic Hippocampus.” Nature Communications 6: 6031. 10.1038/ncomms7031. PMC4627576 25615886

[brb371148-bib-0032] Keezer, M. R. , S. M. Sisodiya , and J. W. Sander . 2016. “Comorbidities of Epilepsy: Current Concepts and Future Perspectives.” Lancet Neurology 15, no. 1: 106–115. 10.1016/S1474-4422(15)00225-2. 26549780

[brb371148-bib-0033] Kumar, P. , A. Lim , S. N. Hazirah , et al. 2022. “Single‐Cell Transcriptomics and Surface Epitope Detection in human Brain Epileptic Lesions Identifies Pro‐inflammatory Signaling.” Nature Neuroscience 25, no. 7: 956–966. 10.1038/s41593-022-01095-5. 35739273 PMC9276529

[brb371148-bib-0034] Kurki, M. I. , J. Karjalainen , P. Palta , et al. 2023. “FinnGen Provides Genetic Insights From a Well‐phenotyped Isolated Population.” Nature 613, no. 7944: 508–518. 10.1038/s41586-022-05473-8. 36653562 PMC9849126

[brb371148-bib-0035] Kwok, M. K. , and C. M. Schooling . 2021. “Herpes Simplex Virus and Alzheimer's Disease: A Mendelian Randomization Study.” Neurobiology of Aging 99: 101.e11–101.e13. 10.1016/j.neurobiolaging.2020.09.025. 33139072

[brb371148-bib-0036] Lee, Y. H. , and G. G. Song . 2019. “Uric Acid Level, Gout and Bone Mineral Density: A Mendelian Randomization Study.” European Journal of Clinical Investigation 49, no. 9:e13156. 10.1111/eci.13156. 31294819

[brb371148-bib-0037] Lilleker, J. B. , J. Vencovsky , G. Wang , et al. 2018. “The EuroMyositis Registry: An International Collaborative Tool to Facilitate Myositis Research.” Annals of the Rheumatic Diseases 77, no. 1: 30–39. 10.1136/annrheumdis-2017-211868. 28855174 PMC5754739

[brb371148-bib-0038] Lundberg, I. E. , M. Fujimoto , J. Vencovsky , et al.2021. “Idiopathic Inflammatory Myopathies.” Nature Reviews Disease Primers 7, no. 1: 86. 10.1038/s41572-021-00321-x. 34857798

[brb371148-bib-0039] Maurer‐Morelli, C. V. , J. F. de Vasconcellos , E. M. Bruxel , et al. 2022. “Gene Expression Profile Suggests Different Mechanisms Underlying Sporadic and Familial Mesial Temporal Lobe Epilepsy.” Experimental Biology and Medicine 247, no. 24: 2233–2250. 10.1177/15353702221126666. 36259630 PMC9899983

[brb371148-bib-0040] McHugh, N. J. , and S. L. Tansley . 2018. “Autoantibodies in Myositis.” Nature Reviews Rheumatology 14, no. 5: 290–302. 10.1038/nrrheum.2018.56. 29674612

[brb371148-bib-0041] Miller, F. W. , J. A. Lamb , J. Schmidt , and K. Nagaraju . 2018. “Risk Factors and Disease Mechanisms in Myositis.” Nature Reviews Rheumatology 14, no. 5: 255–268. 10.1038/nrrheum.2018.48. 29674613 PMC6745704

[brb371148-bib-0042] Nguyen, D. T. , T. M. Le , T. Hattori , et al. 2021. “The ATF6β‐Calreticulin Axis Promotes Neuronal Survival Under Endoplasmic Reticulum Stress and Excitotoxicity.” Scientific Reports 11, no. 1:13086. 10.1038/s41598-021-92529-w. 34158584 PMC8219835

[brb371148-bib-0043] Nissan, E. , A. Watad , A. D. Cohen , et al. 2021. “Epilepsy as a Comorbidity in Polymyositis and Dermatomyositis—A Cross‐Sectional Study.” International Journal of Environmental Research and Public Health 18, no. 8: 3983. 10.3390/ijerph18083983. 33920065 PMC8068784

[brb371148-bib-0044] Ravizza, T. , B. Gagliardi , F. Noé , K. Boer , E. Aronica , and A. Vezzani . 2008. “Innate and Adaptive Immunity During Epileptogenesis and Spontaneous Seizures: Evidence From Experimental Models and Human Temporal Lobe Epilepsy.” Neurobiology of Disease 29, no. 1: 142–160. 10.1016/j.nbd.2007.08.012. 17931873

[brb371148-bib-0045] Ritchie, M. E. , B. Phipson , D. Wu , et al. 2015. “limma Powers Differential Expression Analyses for RNA‐Sequencing and Microarray Studies.” Nucleic Acids Research 43, no. 7:e47. 10.1093/nar/gkv007. 25605792 PMC4402510

[brb371148-bib-0046] Sakaue, S. , M. Kanai , Y. Tanigawa , et al. 2021. “A Cross‐Population Atlas of Genetic Associations for 220 Human Phenotypes.” Nature Genetics 53, no. 10: 1415–1424. 10.1038/s41588-021-00931-x. 34594039 PMC12208603

[brb371148-bib-0047] Sanz, P. , and M. A. Garcia‐Gimeno . 2020. “Reactive Glia Inflammatory Signaling Pathways and Epilepsy.” International Journal of Molecular Sciences 21, no. 11: 4096. 10.3390/ijms21114096. 32521797 PMC7312833

[brb371148-bib-0048] Scheffer, I. E. , S. Berkovic , G. Capovilla , et al. 2017. “ILAE Classification of the Epilepsies: Position Paper of the ILAE Commission for Classification and Terminology.” Epilepsia 58, no. 4: 512–521. 28276062 10.1111/epi.13709 PMC5386840

[brb371148-bib-0049] Shinjo, S. K. , F. H. de Souza , and J. C. de Moraes . 2013. “Dermatomyositis and Polymyositis: From Immunopathology to Immunotherapy (immunobiologics).” Revista Brasileira De Reumatologia 53, no. 1: 101–110. 10.1016/s2255-5021(13)70010-5. 23588520

[brb371148-bib-0050] Sinha, P. , B. Verma , and S. Ganesh . 2021. “Trehalose Ameliorates Seizure Susceptibility in Lafora Disease Mouse Models by Suppressing Neuroinflammation and Endoplasmic Reticulum Stress.” Molecular Neurobiology 58, no. 3: 1088–1101. 10.1007/s12035-020-02170-3. 33094475

[brb371148-bib-0051] Steriade, C. , M. J. Titulaer , A. Vezzani , J. W. Sander , and R. D. Thijs . 2021. “The Association Between Systemic Autoimmune Disorders and Epilepsy and Its Clinical Implications.” Brain: A Journal of Neurology 144, no. 2: 372–390. 10.1093/brain/awaa362. 33221878

[brb371148-bib-0052] Thijs, R. D. , R. Surges , T. J. O'Brien , and J. W. Sander . 2019. “Epilepsy in Adults.” Lancet 393, no. 10172: 689–701. 10.1016/S0140-6736(18)32596-0. 30686584

[brb371148-bib-0053] Verbanck, M. , C. Y. Chen , B. Neale , and R. Do . 2018. “Publisher Correction: Detection of Widespread Horizontal Pleiotropy in Causal Relationships Inferred From Mendelian Randomization Between Complex Traits and Diseases.” Nature Genetics 50, no. 8: 1196. 10.1038/s41588-018-0099-7. 29967445

[brb371148-bib-0054] Verbanck, M. , C. Y. Chen , B. Neale , and R. Do . 2018. “Publisher Correction: Detection of Widespread Horizontal Pleiotropy in Causal Relationships Inferred From Mendelian Randomization Between Complex Traits and Diseases.” Nature Genetics 50, no. 8: 1196. 10.1038/s41588-018-0164-2. 29967445

[brb371148-bib-0055] Vezzani, A. , T. Ravizza , P. Bedner , E. Aronica , C. Steinhäuser , and D. Boison . 2022. “Astrocytes in the Initiation and Progression of Epilepsy.” Nature Reviews Neurology 18, no. 12: 707–722. 10.1038/s41582-022-00727-5. 36280704 PMC10368155

[brb371148-bib-0056] Wu, H. , C. Guo , C. Wang , et al. 2023. “Single‐Cell RNA Sequencing Reveals Tumor Heterogeneity, Microenvironment, and Drug‐Resistance Mechanisms of Recurrent Glioblastoma.” Cancer Science 114, no. 6: 2609–2621. 10.1111/cas.15773. 36853018 PMC10236634

[brb371148-bib-0057] Xu, D. , S. D. Miller , and S. Koh . 2013. “Immune Mechanisms in Epileptogenesis.” Frontiers in Cellular Neuroscience 7: 195. 10.3389/fncel.2013.00195. 24265605 PMC3821015

[brb371148-bib-0058] Xu, S. , E. Hu , Y. Cai , et al. 2024. “Using clusterProfiler to Characterize Multiomics Data.” Nature Protocols 19, no. 11: 3292–3320. 10.1038/s41596-024-01020-z. 39019974

[brb371148-bib-0059] Xue, A. , Z. Zhu , H. Wang , et al. 2024. “Unravelling the Complex Causal Effects of Substance Use Behaviours on Common Diseases.” Communications Medicine (Lond) .4, no. 1: 43. 10.1038/s43856-024-00473-3. PMC10933313 38472333

