# Supplementary material for: Genetic Association Between Polymyositis/Dermatomyositis and Epilepsy: Insights From Mendelian Randomization and Bioinformatic Analyses
Source: Brain Behav. 2025 Dec 29;16(1):e71148. doi: 10.1002/brb3.71148 (PMC12748525; doi:10.1002/brb3.71148)
Supplement: Supplementary file 10 — Figures S1–S6 [file BRB3-16-e71148-s004.docx]

**Supporting information**

| **A**  **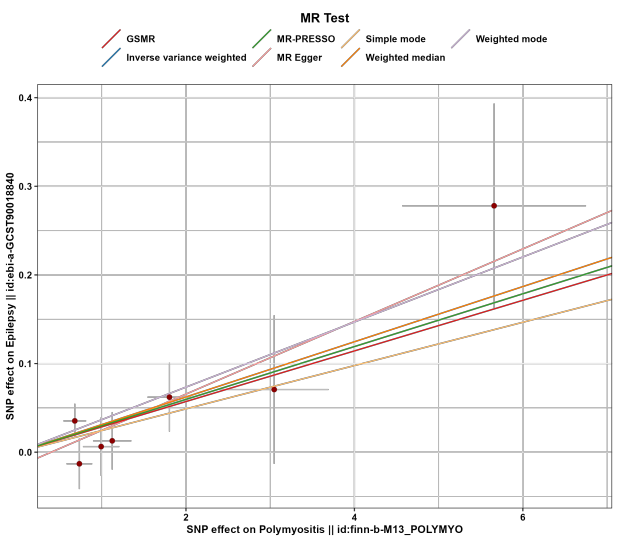** | **B**  **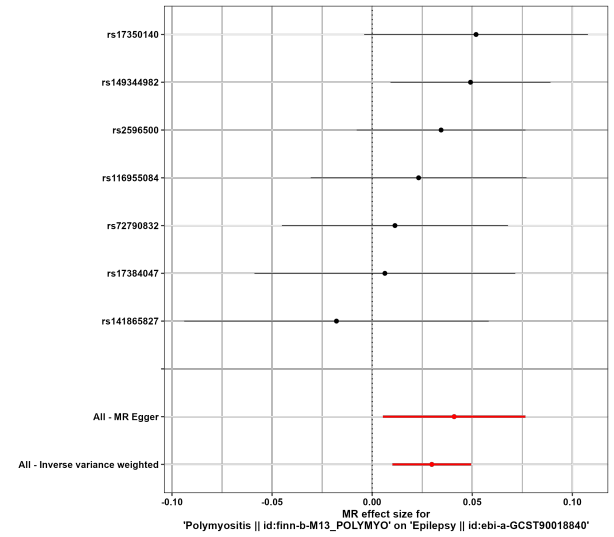** |
| --- | --- |
| **C**  **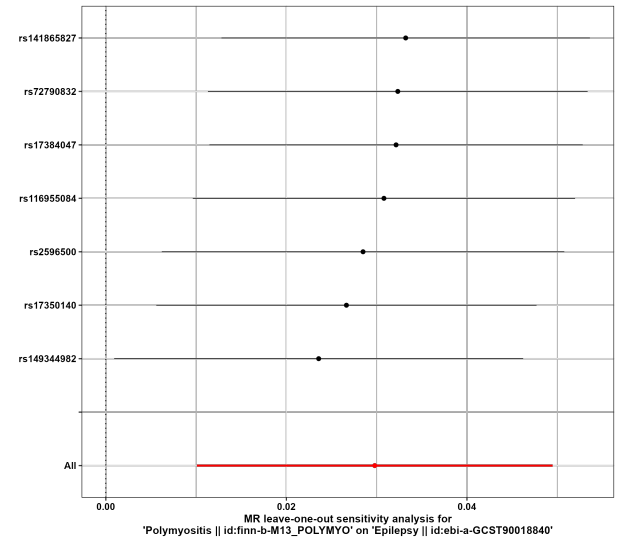** | **D**  **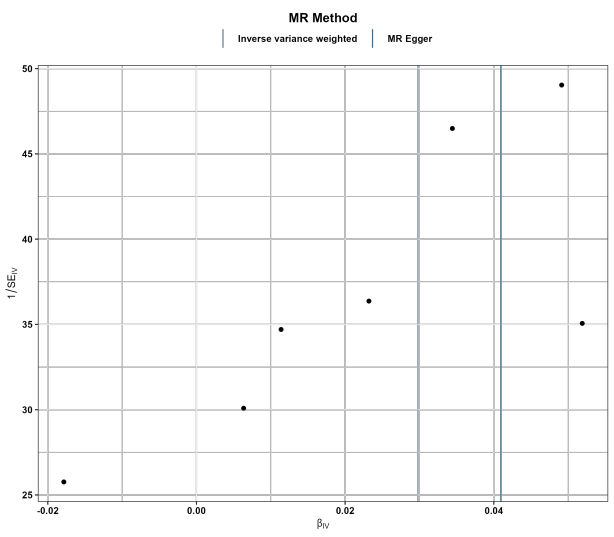** |

**Figure S1.** Mendelian Randomization Analysis of Polymyositis (exposure) on Epilepsy. A-D. scatter plot, Forest plot,"Leave-one-out" analysis scatter plot and funnel plot of Polymyositis, and epilepsy.

| **A**  **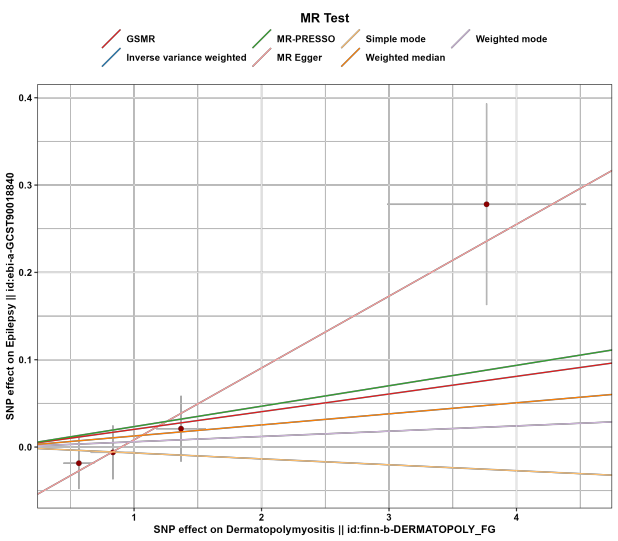** | **B**  **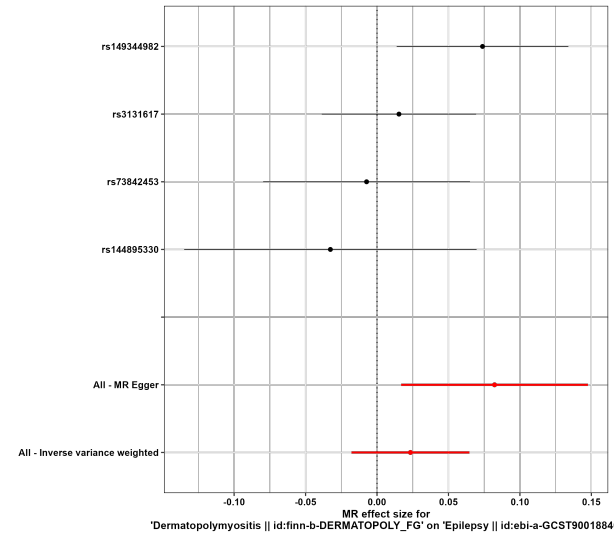** |
| --- | --- |
| **C**  **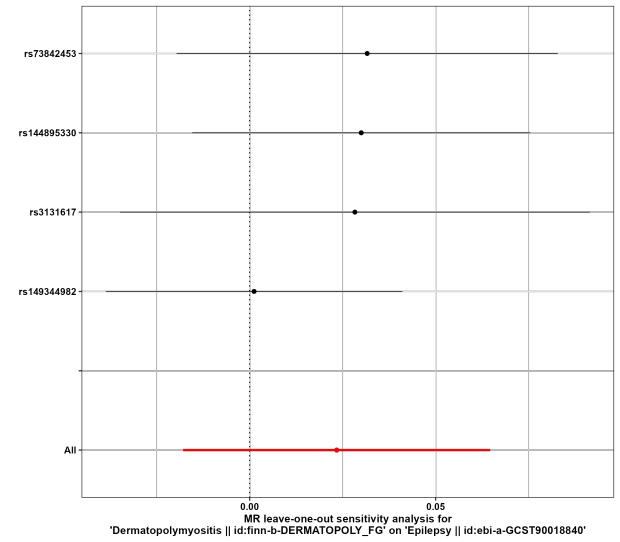** | **D**  **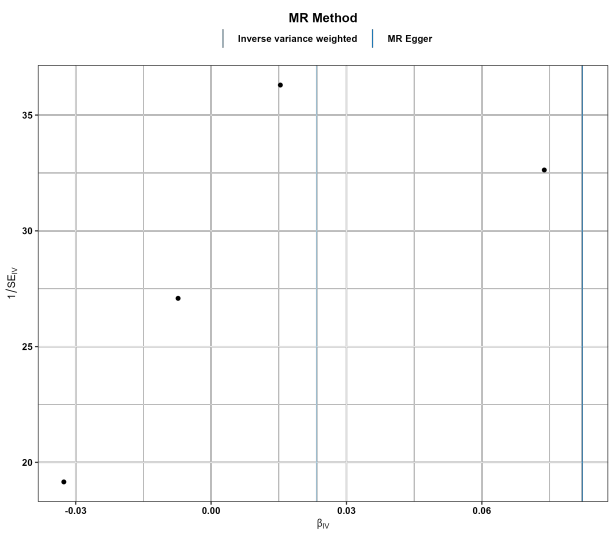** |

**Figure S2. Mendelian Randomization Analysis of Dermatomyositis(exposure) on epilepsy.**

| **A**  **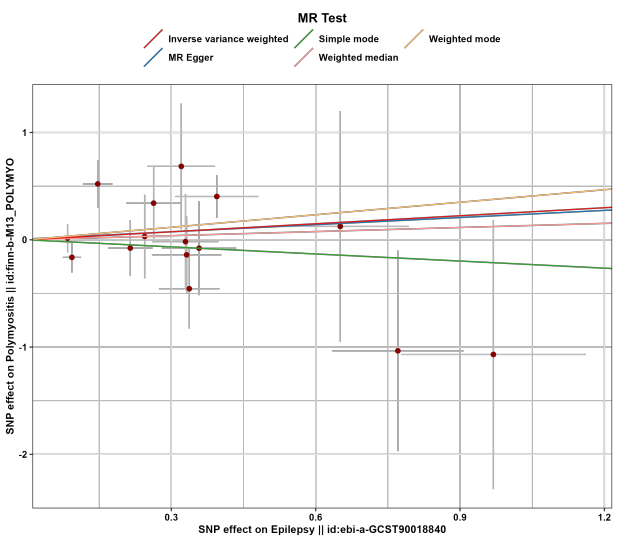** | **B**  **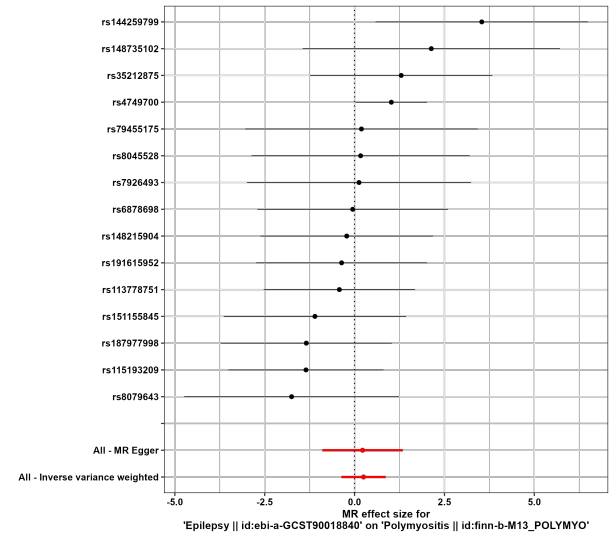** |
| --- | --- |
| **C**  **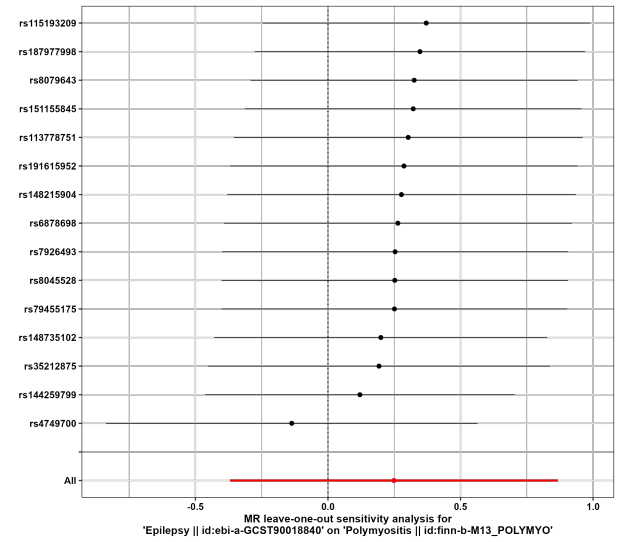** | **D**  **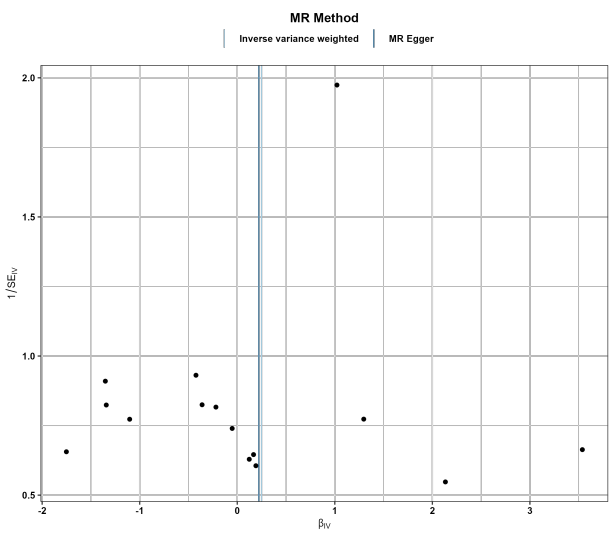** |

**Figure S3. Mendelian Randomization Analysis of epilepsy(exposure) on Polymyositis.**

| **A**  **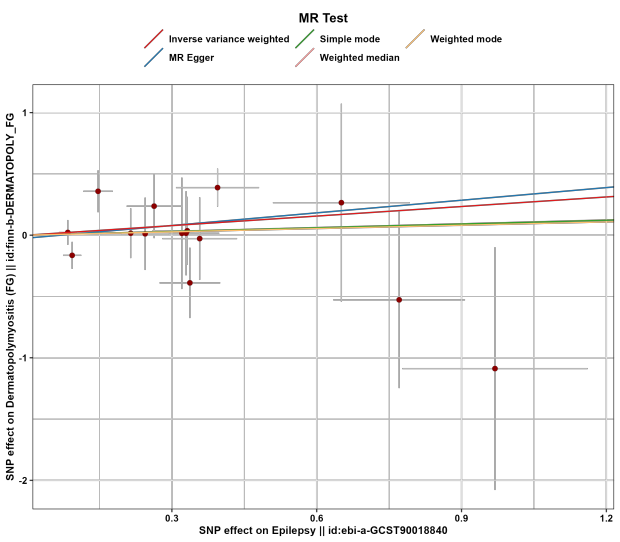** | **B**  **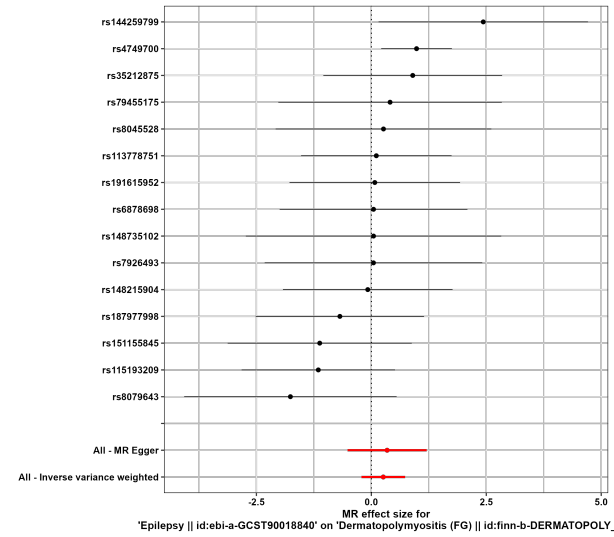** |
| --- | --- |
| **C**  **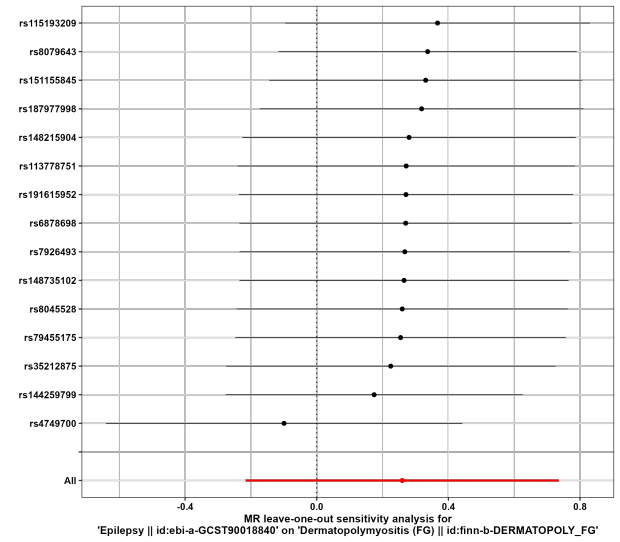** | **D**  **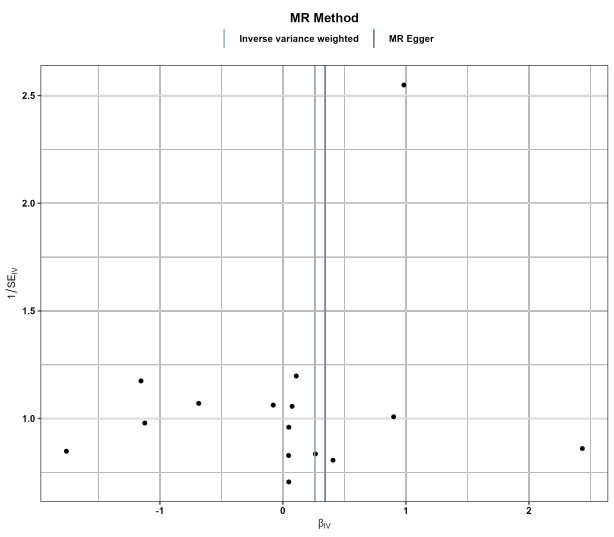** |

**Figure S4. Mendelian Randomization Analysis of epilepsy(exposure) on Dermatomyositis.**

**
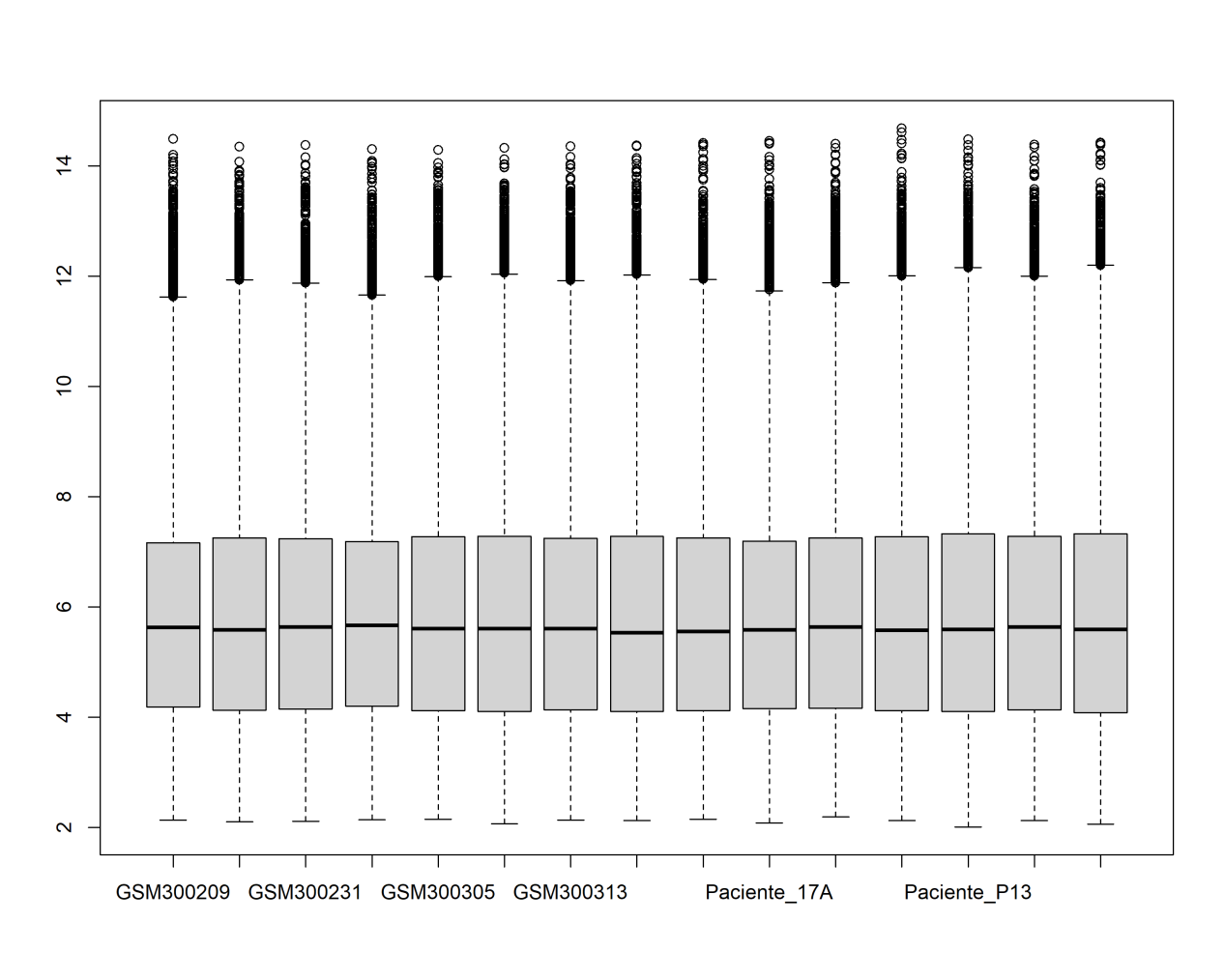
**

**Figure S5. The boxplot illustrates the distribution of gene expression values across samples in the GSE202101 dataset.**

**
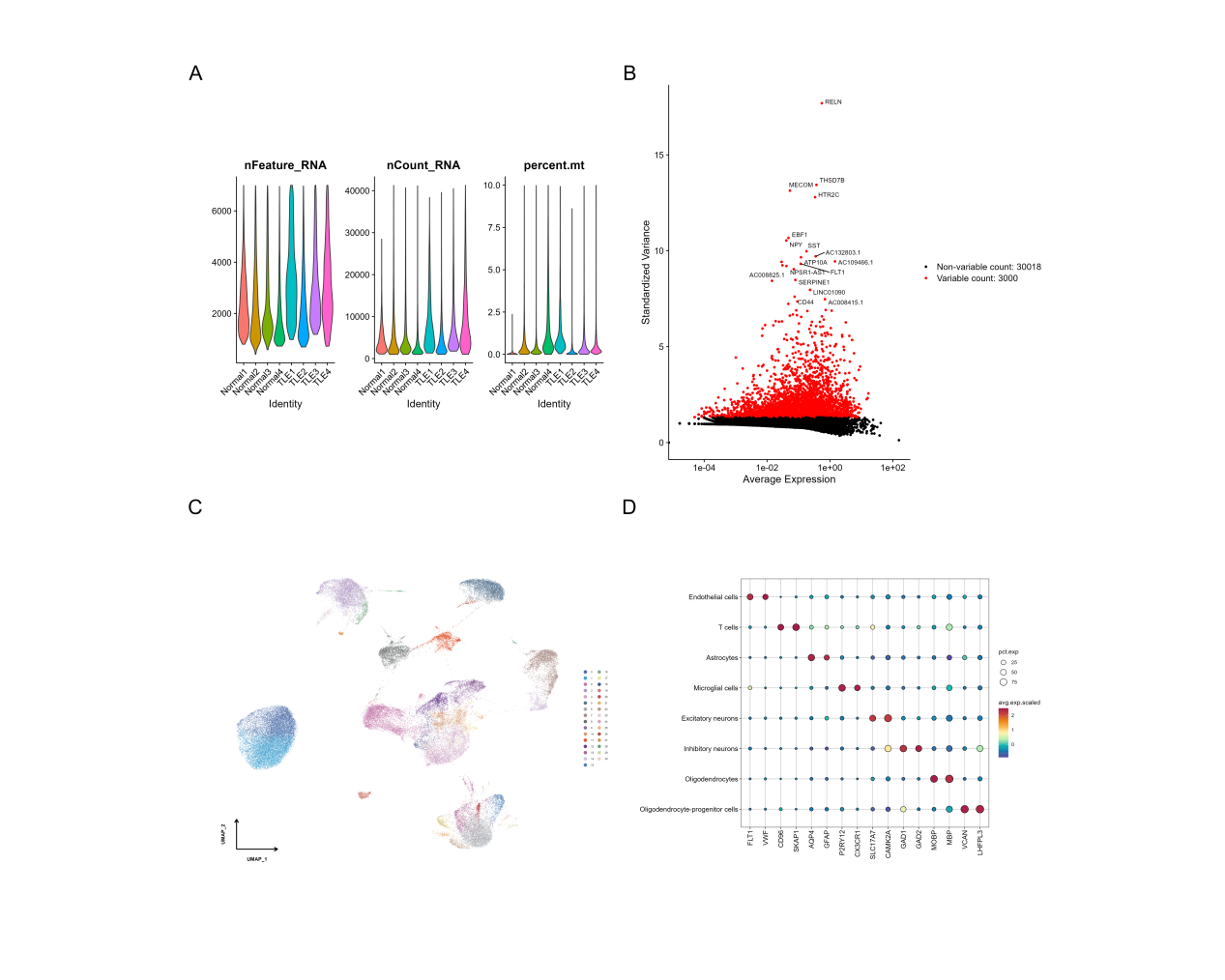
**

**Figure S6. Single cell RNA-seq data analysis.** (A) The distribution plot of the number of unique genes, UMI count content and mitochondrial gene expression ratio in each cell of each samplee. (B) The scatter plot of highlyFudanvariable genes.(C) The diagram of Uniform Manifold Approximation and Projection (umap) distribution in samples of different cluster.(D)Dot plot depicting selected differentially expressed genes for each cluster. Dot size corresponds to the percentage of nuclei expressing the gene in each cluster; color represents the average gene expression level.
