# Supplementary figures and images for: Genetic Association Between Polymyositis/Dermatomyositis and Epilepsy: Insights From Mendelian Randomization and Bioinformatic Analyses
Source: Brain Behav. 2025 Dec 29;16(1):e71148. doi: 10.1002/brb3.71148 (PMC12748525; doi:10.1002/brb3.71148)

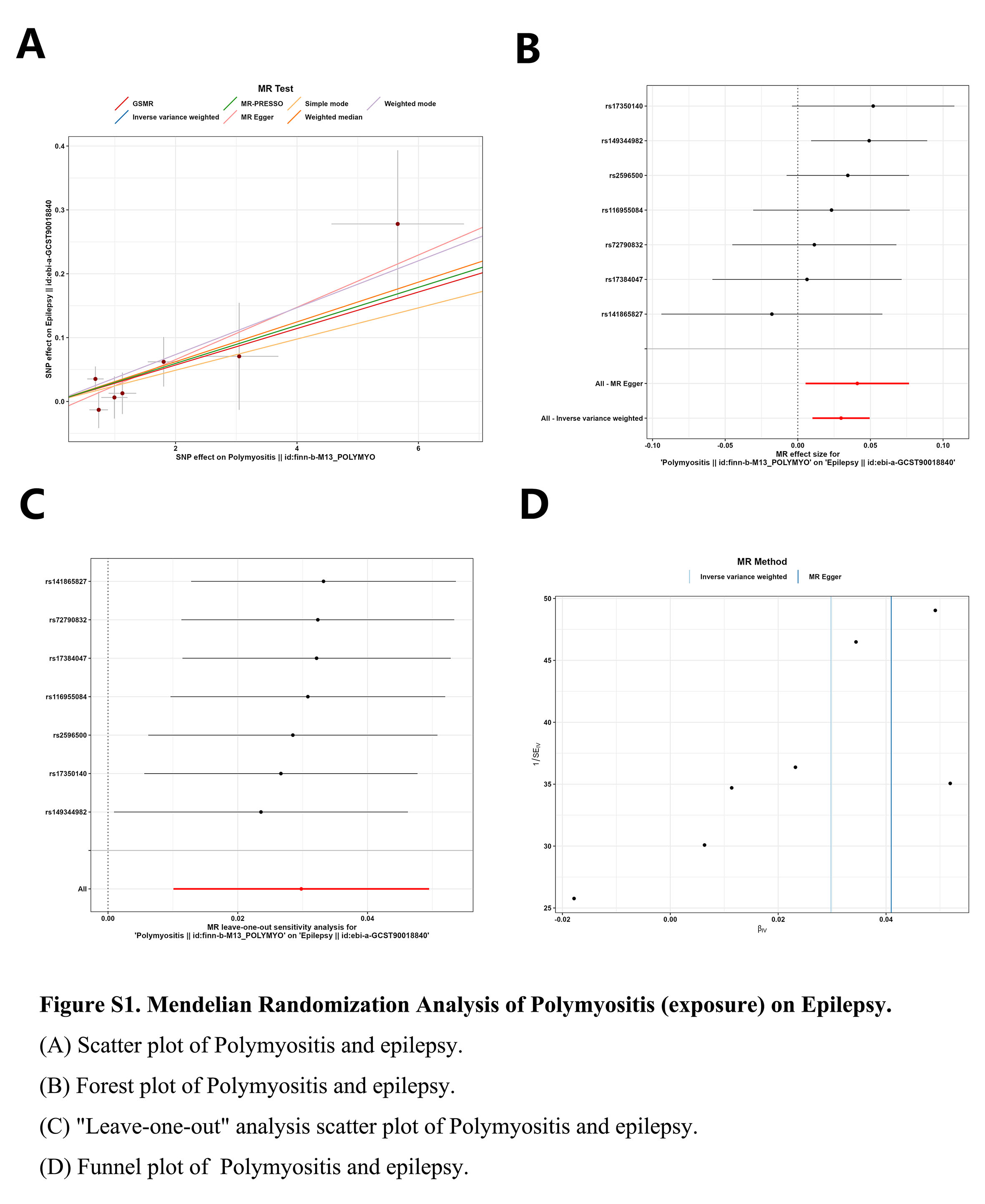

Supplement: Supplementary file 11 — Figure S1 The Mendelian Randomization Analysis of Polymyositis (exposure) on Epilepsy. [file BRB3-16-e71148-s005.jpg]

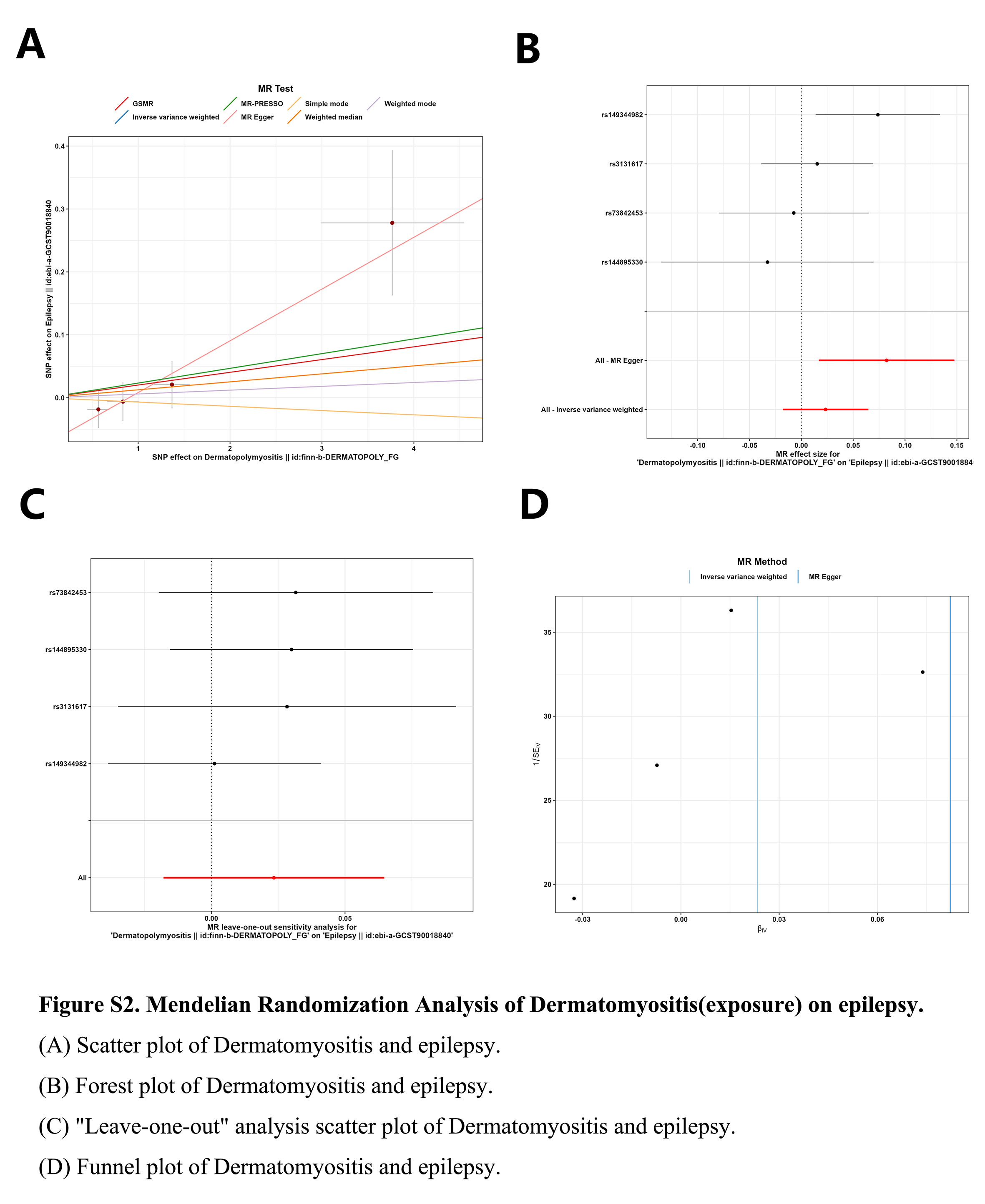

Supplement: Supplementary file 12 — Figure S2 The Mendelian Randomization Analysis ofDermatomyositis(exposure) on epilepsy. [file BRB3-16-e71148-s006.jpg]

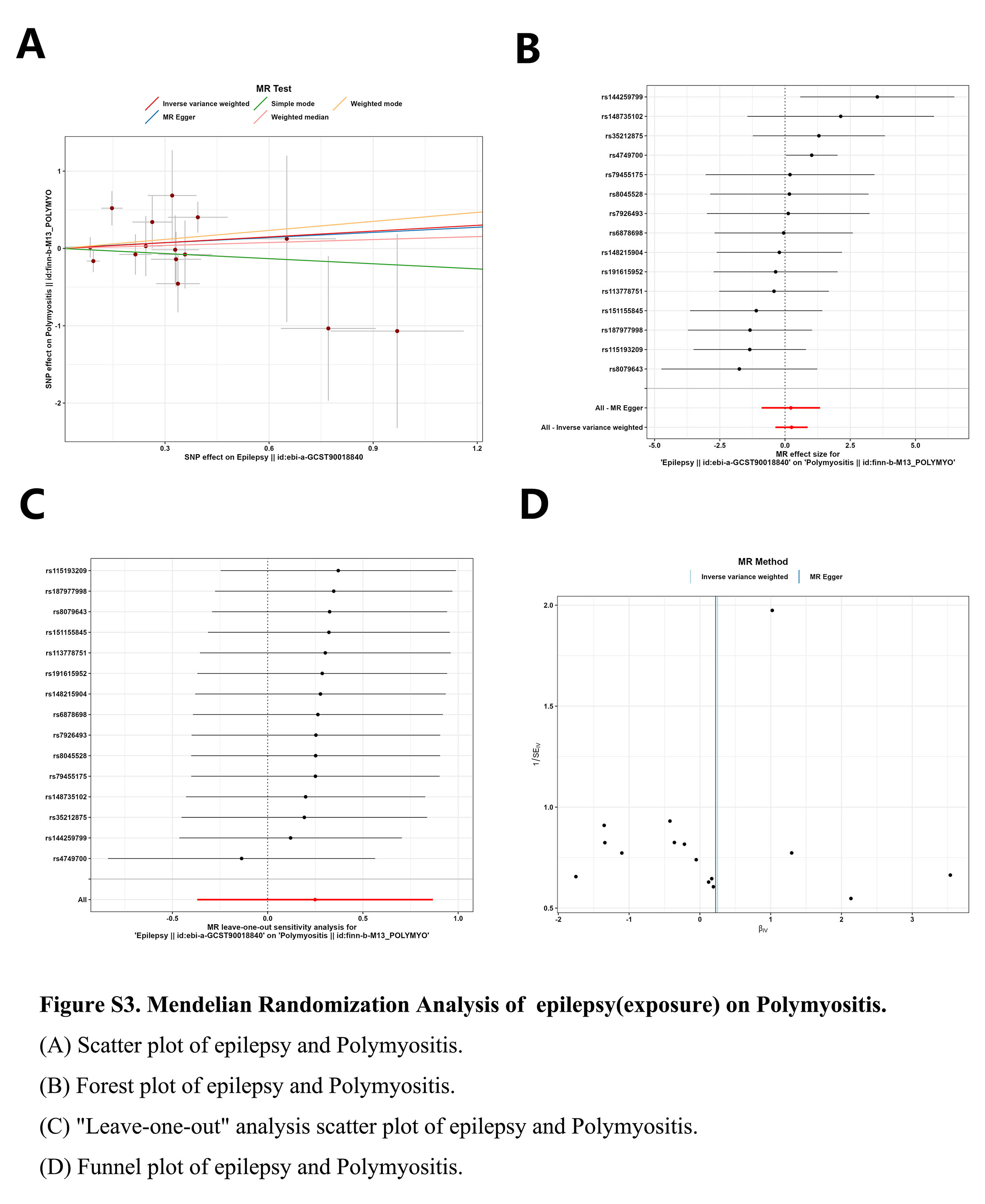

Supplement: Supplementary file 13 — Figure S3 The Mendelian Randomization Analysis ofepilepsy(exposure) on Polymyositis. [file BRB3-16-e71148-s015.jpg]

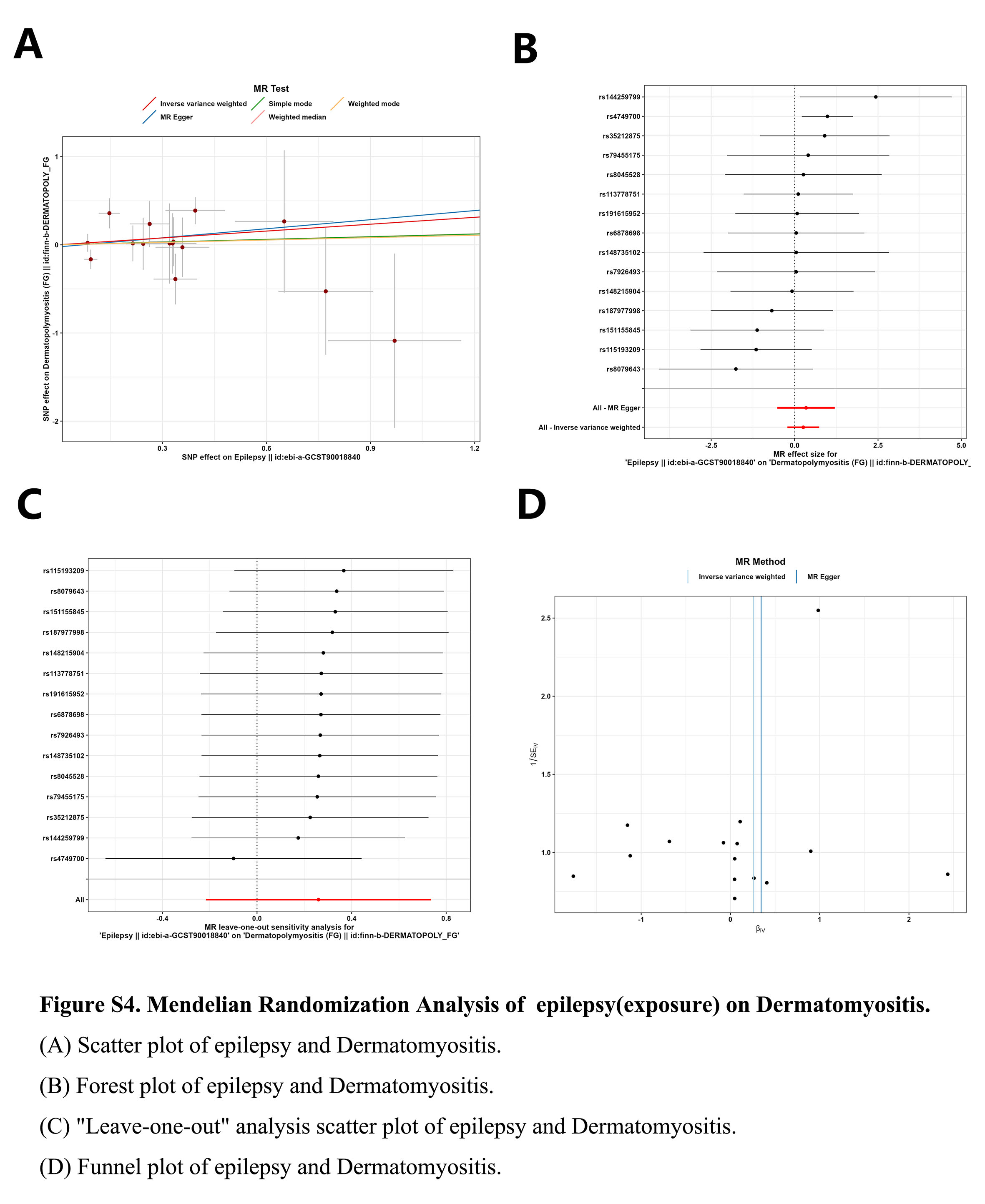

Supplement: Supplementary file 14 — Figure S4 The Mendelian Randomization Analysis ofepilepsy(exposure) on Dermatomyositis. [file BRB3-16-e71148-s014.jpg]

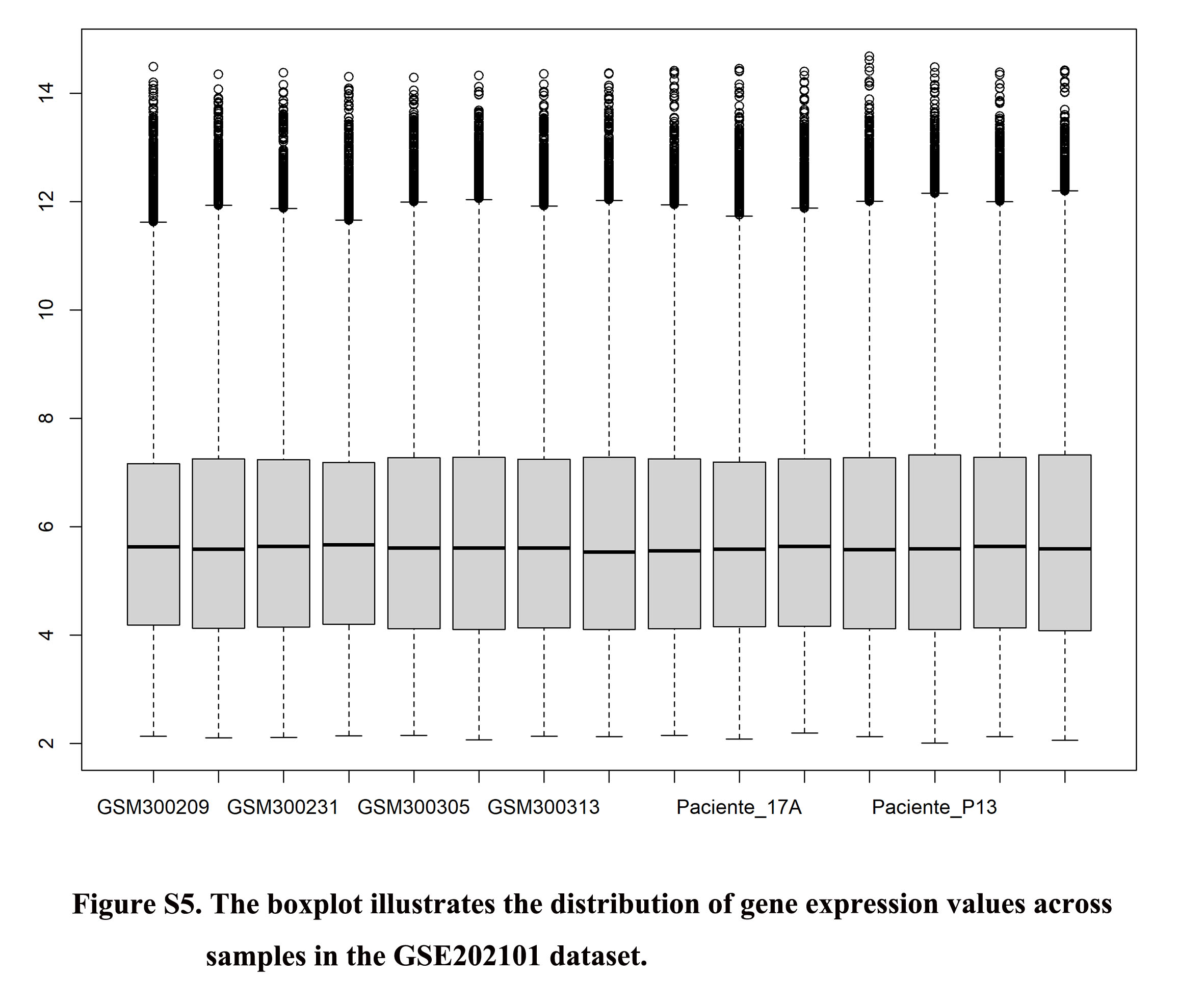

Supplement: Supplementary file 15 — Figure S5 The boxplot illustrates the distribution of gene expression values across samples in the GSE202101 dataset. [file BRB3-16-e71148-s012.jpg]

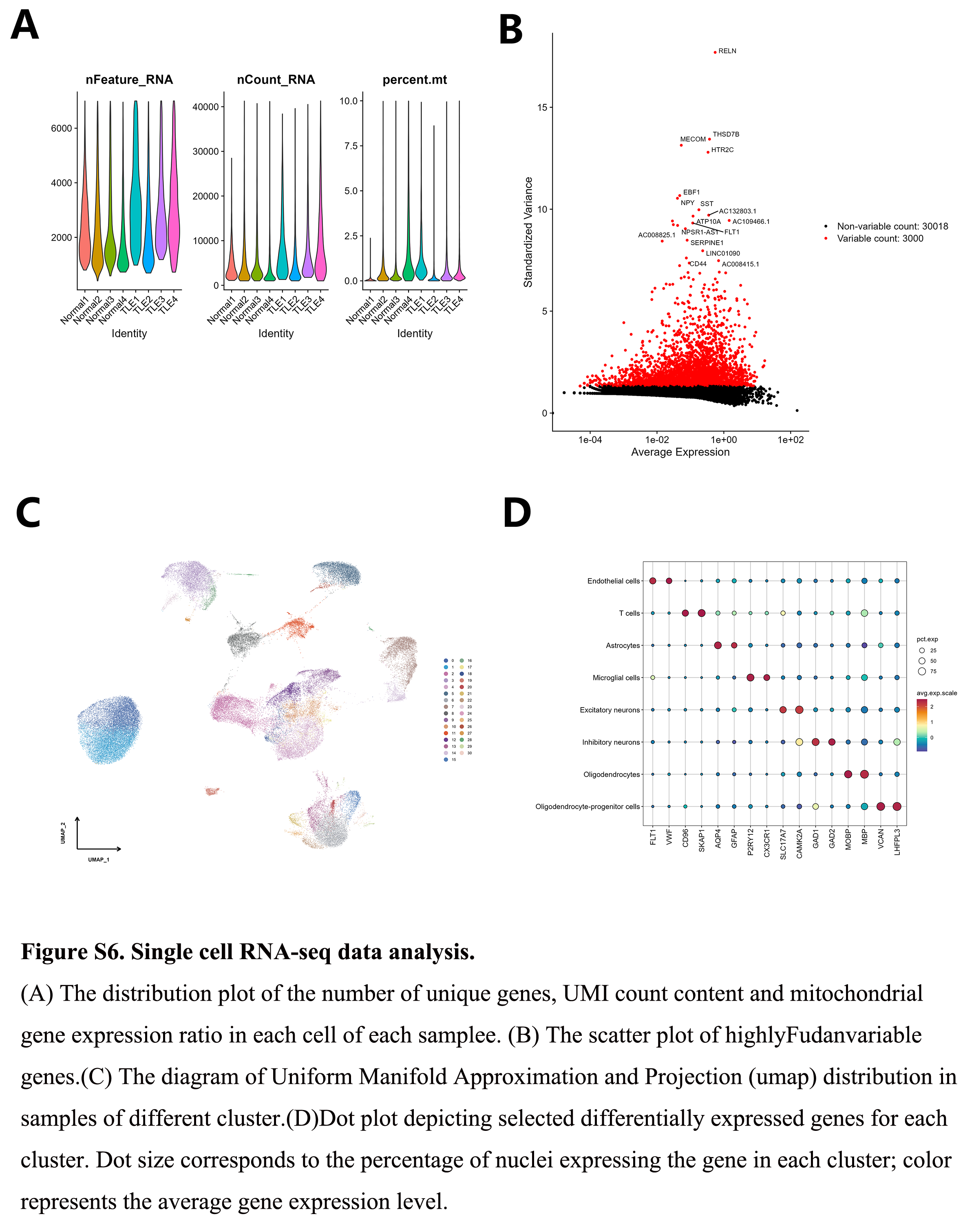

Supplement: Supplementary file 16 — Figure S6 The Single cell RNA‐seq data analysis. [file BRB3-16-e71148-s008.tif]
